# CUL7-mediated KEAP1 ubiquitination promotes the progression of colon cancer via NRF2 signaling

**DOI:** 10.1038/s41419-026-08929-1

**Published:** 2026-06-08

**Authors:** Le Shi, Feiyu Shi, Yue Zhang, Ruichen Liu, Quanyu Liu, Shuangxi Zhang, Zhen Wang, Wu Su, Dan Gao, Yi Liu, Xiangpeng Dai, Hui Guo, Jiankang Liu, Junjun She, Jiangang Long

**Affiliations:** 1 Center for Mitochondrial Biology and Medicine, The Key Laboratory of Biomedical Information Engineering of Ministry of Education, School of Life Science and Technology, Xi’an Jiaotong University, Xi’an, China; 2 Department of General Surgery, The First Affiliated Hospital of Xi’an Jiaotong University, Xi’an, China; 3 Institute of Molecular and Translational Medicine, School of Basic Medical Sciences, Xi’an Jiaotong University Health Science Center, Xi’an, China; 4 Department of Oncology, Shaanxi Provincial People’s Hospital, Xi’an, China; 5 Key Laboratory of Organ Regeneration and Transplantation of Ministry of Education, First Hospital, Jilin University, Changchun, China; 6 Department of Endocrinology, The First Affiliated Hospital of Medical College, Xi’an Jiaotong University, Xi’an, China; 7 School of Life Sciences and Health, University of Health and Rehabilitation Sciences, Qingdao, China; 8 Department of Dermatology, The First Affiliated Hospital of Medical College, Xi’an Jiaotong University, Xi’an, China; 9 Department of High Talent, The First Affiliated Hospital of Medical College, Xi’an Jiaotong University, Xi’an, China; 10 Shaanxi Belt and Road Joint Laboratory on Gut Microbiome and Cancer, Yulin, China

**Keywords:** Colon cancer, Ubiquitylation

## Abstract

Cullin7 (CUL7) mediates cancer progression across multiple cancer types through its regulatory role in protein ubiquitination. However, the biological function and molecular mechanisms underlying CUL7 in colon cancer remain poorly defined. Here, we demonstrate that high CUL7 expression correlates with poor prognosis in patients. Ablation of *CUL7* inhibited cell proliferation and migration in colon cancer cells, whereas CUL7 overexpression exhibited oncogenic properties. Moreover, *Cul7*-deplete mice exhibit a lower level of tumor growth. Mechanistically, CUL7 interacts with Kelch-like ECH-associated protein 1 (KEAP1) and catalyzes K29- and K48-linked polyubiquitination to promote its proteasomal degradation, which is crucial for NRF2 signaling. This leads to the decline of reactive oxygen species (ROS) and promotion of cancer growth. Importantly, the C-terminus of CUL7 is a crucial for governing KEAP1 stability and orchestrating antioxidant defense and cell growth. Clinical analysis identifies an inverse correlation between CUL7 and KEAP1 expression, and a positive correlation between CUL7 and NRF2 levels in human colon cancer. Our findings indicate that CUL7 mediates NRF2 signaling through promoting the KEAP1 ubiquitination, a mechanism that is integral to colon cancer progression. Collectively, these results establish CUL7 as a potential therapeutic target for colon cancer.

## Introduction

Colorectal cancer (CRC) is one of the most prevalent malignancies, ranking third in incidence and second in mortality worldwide [1]. Although significant progress has been made in therapeutic modalities, including surgery, radiotherapy, and chemotherapy, the prognosis for CRC remains suboptimal, largely due to late-stage diagnosis, metastasis, and immune evasion [2]. Therefore, it is essential to elucidate the key signaling pathways implicated in the CRC progression, identify potential therapeutic targets, and improve diagnostic and intervention strategies.

The ubiquitin-proteasome system (UPS), a critical type of post-translational modification (PTM), serves as a fundamental regulatory system orchestrating diverse cellular processes, including signal transduction, metabolic regulation, development, apoptosis, cell cycle, and protein quality control. Dysregulation of the UPS has been extensively implicated in the pathogenesis of various human disease, such as neurodegeneration, inflammation, and cancer [3]. In cancer therapy, targeting specific components of UPS, particularly E3 ubiquitin ligases, have emerged as a promising strategy, as inhibitors of E1 or E2 enzymes have shown limited clinical efficacy and have not advanced significantly in clinical trials [4, 5]. Among E3 ubiquitin ligases, RING ligases constitute the largest subclass, with Cullin-RING ligases (CRLs) composed of Cullin (CUL) proteins, including CUL1, CUL2, CUL3, CUL4A, CUL4B, CUL5, and CUL7. These ligases are responsible for approximately 20% of cellular protein ubiquitination and subsequent degradation [6, 7]. Notably, CUL7 exhibits unique features compared with other CUL family members, primarily due to its distinct motifs, namely the DOC and CPH domains. While FBXW8 (also known as FBX29, FBW6, or FBW8) was initially identified as the exclusive F-box protein interacting with CUL7 via SKP1, recent biochemical studies have revealed that CUL7 can form complexes with other adaptors independent of FBXW8 [8, 9]. Originally associated with genetic disorders such as 3-M syndrome and Yakut short stature syndrome through loss-of-function mutations, CUL7 has subsequently been characterized as a potentially oncogenic driver in multiple malignancies [10]. Nevertheless, the biological functions and molecular mechanisms of CUL7 in different cancer types remains poorly understood. In CRC, studies have consistently demonstrated that CUL7 expression is significantly upregulated in tumor tissues compared with adjacent normal tissues. Notably, high CUL7 expression-but not that of other CUL family members-correlates with poor survival outcomes in colon cancer patients [11–13]. These findings suggest a specific role of CUL7 in CRC progression. However, the biological function and molecular mechanisms underlying CUL7-mediated progression in CRC remain elusive, and the identification of its downstream targets and signaling is crucial for understanding its oncogenic functions and developing targeted therapies.

In this study, through integrated CoIP and proteomic profiling analyses, Kelch-like ECH-associated protein 1 (KEAP1) emerged as the predominant candidate target of CUL7-mediated ubiquitination. KEAP1, an adaptor protein for NRF2, governs the ubiquitination of NRF2, a master transcriptional regulator of cellular redox homeostasis [14, 15]. Intriguingly, KEAP1 itself is also subject to ubiquitination [16, 17], and its ubiquitinated form can be degraded via both autophagy [18] and the proteasome pathway [19], thereby contributing to redox homeostasis. We further performed a series of functional assays to illustrate that CUL7 promotes colon cancer progression by catalyzing K29- and K48-linked polyubiquitination of KEAP1, targeting it for proteasomal degradation. This CUL7-KEAP1 axis potentiates NRF2 signaling and cellular survival. Collectively, these findings provide essential insights into the oncogenic properties and molecular network of CUL7 in colon cancer.

## Materials and methods

### Clinical specimens

Colon cancer tissues and adjacent normal tissues were gathered from patients (*n* = 12) who underwent surgery in the Department of General Surgery, The First Affiliated Hospital of Xi’an Jiaotong University.

### Animals

The Cre/loxP system was employed to achieve tissue-specific transgenic mice. *Cul7*-floxed mice (homozygous, *Cul7*^*fl/fl*^, Heterozygous, *Cul7*^*fl/-*^) were generated by Cyagen Biosciences (Beijing, China). Briefly, two sgRNAs were designed to mediate the insertion of two loxP sites franking a ~ 5 kb chromosomal region (exon 2–9) at the *Cul7* locus in the mouse genome. Intestinal-specific *Cul7* knockout mice (*CUL7*^*fl/fl*, *Vil-cre*^) were established by crossing *Cul7*^*fl/fl*^ or *Cul7*^*fl/-*^ with *Cul7*^*fl/-, Vil-cre*^ mice, with littermates *Cul7*^*fl/fl*^ mice serving as control group. Transgenic mice were identified by PCR of tail-tip and colon genomic DNA. For the azoxymethane/dextran sulphate-sodium (AOM/DSS, AD) mice model, eight-week-old mice were injected intraperitoneally with AOM (10 mg/kg body weight). Seven days after AOM injection, the mice were given water containing 2.5% DSS for seven days, followed by regular drinking water for the next two weeks. DSS treatment was given for an additional two cycles, with the mice being euthanized on day 76. The mice were randomly assigned to four groups: *Cul7*^*fl/fl*^+Water (*n* = 5), *Cul7*^*fl/fl*^ + AD (*n* = 6), *Cul7*^*fl/fl*, *Vil-cre*^+Water (*n* = 5), *Cul7*^*fl/fl*, *Vil-cre*^ + AD (*n* = 8).

In xenograft experiments, female BALB/c nude mice aged 5 weeks were purchased from GemPharmatech Co., Ltd (Chengdu, China). In brief, 5 × 10^6^ HCT116 cells or 8 × 10^6^ DLD1 cells in 100 μL PBS were subcutaneously injected into the flank of nude mice. The tumor sizes were measured every 7 or 5 days with a vernier caliper and tumor volume was calculated as V = (length x width^2^)/2. 4 weeks post injection, mice were sacrificed and solid tumors were dissected, weighed and collected for further analysis. The mice were randomly assigned to two or three groups: shGFP and sh*CUL7* or shGFP, sh*CUL7* and sh*CUL7*+sh*KEAP1*.

### Antibodies and reagents

The primary antibody used were purchased from the following suppliers: sigma (anti-CUL7, C1743, 1:1000); Abcam (anti-CUL7, ab115304, 1:3000); Cell Signaling Technology (anti-KEAP1, #4678, 1:1000; anti-GAPDH, #2118, 1:1000; anti-α-tubulin, #2144, 1:1000); Proteintech (anti-KEAP1, 10530-2-AP, 1:1000; anti-Flag, 20543-1-AP, 1:1000; anti-Myc, 16286-1-AP, 1:1000; anti-HA, 51064-2-AP, 1:1000; anti-Gst, 10000-0-AP, 1:1000); Santa Cruz (anti-Ub, sc-8017, 1:5000; anti-CUL7, sc-53810,1:1000; anti-FBXW8, sc-514385, 1:1000; anti-Histone, sc-8030, 1:1000; anti-Nrf2, sc-365949); Abclonal (anti-CUL3, A16455, 1:1000; anti-k29-linkage Specific Polyubiquitin Rabbit pAb, A18198, 1:1000; anti-k48--linkage Specific Polyubiquitin Rabbit pAb, A18163, 1:1000); Servicebio (Ki67, GB121141, 1:600; cleaved Caspase-3, GB115733, 1:800; E-cadherin, GB12083, 1:4000). The secondary antibodies were purchase from Jackson lab (anti-mouse IgG, 115-035-003, 1:5000; anti-rabbit IgG, 111-035-003, 1:5000); from Invitrogen (anti-mouse IgG, A21202, 1:100; anti-rabbit IgG, A0468,1:100). Protein A + G Agarose (P2012) was purchased from Beyotime (Shanghai, China). MG132 (BML-PI102-0005) was purchased from Enzo life science (NY, USA). Cycloheximide (CHX, HY12320) was purchased from MedChemExpress. H_2_DCF-DA (D399) was purchased from Life Technologies (San Diego, CA, USA). AOM (0218397125) and DSS (0216011080) were purchased from MP Biomedicals (Santa Ana, CA, USA).

### Plasmids

CUL7 (wild type [WT] and truncation: 1-2577, 1687-2900, 2911-5097, 4501-5097) were amplified and cloned into the pcDNA3.1 vector with an N-terminal Myc tag via HindIII and NotI sites. KEAP1 (WT and truncation: 1-537, 538-945, 961-1872) were also amplified and cloned into the pcDNA3.1 vector with a C-terminal 3xFlag tag via HindIII and XhoI sites. His-Ub and its mutants have been previously described [20]. The sequences for siRNA and shRNA were detailed in Table S1. All plasmids were extracted by using NucleoBond Xtra Midi EF kit according to the manufacturer’s instructions (NMG, Germany).

### Cell culture, cell transfection and viral infection

HCT116, DLD1, HEK293 and HEK293T cells were cultured in Dulbecco’s modified Eagle medium (DMEM) (Corning) containing 10% fetal bovine serum (FBS) (Biological Industries), 100 units of penicillin, and 100 mg/ml streptomycin with 5% CO_2_ at 37°C. All cells were authenticated by short tandem repeats (STR) profiling.

For construction transfection, cells with 60%-80% confluence were transfected using Lipofectamine 2000 (Invitrogen, CA, USA). For siRNA transfection, cells were transfected using Lipofectamine RNAiMAX Transfection Reagent (Invitrogen). Both transfections were conducted according to the manufacturers’ instructions, and cells were harvested and subjected to various assays after transfection for 48 h. For generating stable cell lines, lentivirals carrying shRNA targeting *CUL7*, *KEAP1* or *NRF2* were transfected into 293 T cells with pVSVG and pD8.9 using Lipofectamine 2000. The media containing the viruses were collected at 48 h and 72 h after transfection, and filtered through a 0.45 μm filter. HCT116 and DLD1 cells with 50% confluence were infected with the virus media added with 4 μg/mL polybrene (Sigma). After 48 h infection, cells were selected using 1 or 2 μg/mL puromycin (Sigma) for at least 72 h to eliminate the uninfected cells.

### Quantitative proteomics analysis

HCT116 cells transfected with NC and si*CUL7* were lysed in Western and IP lysis buffer, and the concentration were detected with a BCA Protein Assay kit. Label-free quantitative proteomics analysis was performed by Novogene Bioinformatics Technology Co Ltd. (Beijing, China). Briefly, the proteins were digested with trypsin (Promega), desalted with methanol, formic acid and acetonitrile, and peptide samples were analyzed using Q Exactive^TM^ HF-X MS (Thermo Fisher Scientific). Then, the raw data were analyzed using the UniProt database for *Homo Sapiens* by the search engine Proteome Discoverer 2.2 (Thermo Fisher Scientific). For differential protein expression analysis, peptides were set to 1%, and T-*t*est analysis was used.

### RNA isolation and RT-qPCR

Total RNA was isolated from HCT116 and DLD1 cells with RNAiso Plus (Accurate Biology) and reverse-transcribed into cDNA with Evo M-MLV RT Premix (Accurate Biology), followed by qPCR with AG SYBR and specific primers in BioRad CFX96 qPCR Systems. The 2-△△Ct method was used to analyze the data, with the housekeeping gene GAPDH used as a loading control. The primers related to *CUL7*, *KEAP1*, *NQO1*, *HO-1*, *GAPDH* were listed in Table S2.

### Immunoblot (IB) and immunoprecipitation (IP)

Cells and tissues were washed with PBS and lysed in Western and IP lysis buffer (Beyotime) supplemented with 1 mM PMSF. The total protein concentration were detected with a BCA Protein Assay kit (Pierce), mixed with loading buffer (P1040) and boiled at 100 °C for 10 min. For endogenous immunoprecipitation, the cell lysates were incubated with CUL7 antibody (1 μg), KEAP1 antibody (2 μg) or IgG (1 μg) in a rotating incubator overnight at 4 °C, and then pulled down with protein A/G agarose beads for 1 h. For exogenous immunoprecipitation, 1 mg cell lysates were incubated with beads-conjugated anti-Flag/anti-Myc antibody (1–2 μg) overnight at 4 °C. The immunocomplexes were washed four times with NETN buffer (20 mM Tris, pH 8.0, 100 mM NaCl, 1 mM EDTA, 0.5% NP-40), resolved with SDS-PAGE and analyzed with indicated antibody by western blotting.

### Mass spectrometry (MS) analysis

HEK293T cells were transiently transfected with Myc-CUL7 for 48 h and immunoprecipitated with Myc Tag Immunomagnetic beads (TargetMol). The conjugated proteins were eluted by SDS-PAGE and observed with Coomassie blue stain. The digested gel fractions were processed for LC-MS/MS analysis on a timsTOF Pro2 mass spectrometer (Bruker). MS was performed at Metware Biotechnology Inc. (Wuhan, China), and the data were analyzed via DIA-NN (v1.8.1).

### Protein half-life analysis

Cells were transfected with CUL7 emptor vector (EV), WT and truncations, or NC and si*CUL7*. After 36 h, the cells were treated with the protein synthesis inhibitor CHX (200 μg/mL, Sigma) for different times. Cells were harvested for SDS-PAGE and further incubated with the indicated antibodies. KEAP1 protein band densities were quantified by Quantity One software and normalized to GAPDH.

### In vivo ubiquitination assay

Cells were transfected with CUL7 and its truncations, KEAP1, Ub and its mutants with Lipofectamine 2000. 48 h later, the cells were treated with 10 μM MG132 overnight, washed with PBS and lysed in buffer A (6 M guanidine-HCl, 0.1 M Na_2_HPO_4_/NaH_2_PO_4_, and 10 mM imidazole [pH 8.0]). After sonication, the lysates were centrifuged and incubated with Ni-NTA beads (QIAGEN) for 3 h at room temperature. Then the beads were washed two times with buffer A, two times with buffer A/buffer TI (vol:vol = 1:3), and one time with buffer TI (25 mM Tris-HCl and 20 mM imidazole [pH 6.8]). The protein were released from the Ni-NTA beads by adding 2x protein loading buffer and boiling for 10 min. Pull-down proteins were analyzed by immunoblotting using anti-Flag antibody.

For endogenous ubiquitination of KEAP1 in HCT116 and DLD1 cells, the cells were lysed and processed as described in IB and IP. The protein were resolved with SDS-PAGE and analyzed with anti-Ub antibody by western blotting.

### MTT assay

Cells were transfected with CUL7 WT and its truncation or si*CUL7*. 36 h later, 4 × 10^3^ cells per well (100 μL cell suspensions) were plated in 96-well plates. After 24, 48, 72, 96 h culture, MTT (5 mg/mL) was added to each well and incubated at 37°C for 4 h. Then, the absorbance of each well was measured at 490 nm, and the values were normalized to wells with media alone.

### Colony formation assay

CUL7 WT and truncation or si*CUL7* were transfected into cells. 36 h later, 2000 cells per well for HCT116 cells or 1000 cells per well for DLD1 cells were plated in 6-well plates. The cells were incubated at 37 °C and 5% CO_2_ for growing 10–14 d, stained with crystal violet, and the number of colonies was counted.

### Migration assay

CUL7 WT and truncation, or si*CUL7* and si*KEAP1* were transfected into cells. 36 h later, 1 × 10^5^ cells in serum-free culture medium were added to the 24 cell culture inserts (Corning). The inserts were placed into a 24-well plates pre-added with 600 μL culture medium containing 10% FBS. After 24 h for DLD1 cells or 48 h for HCT116 incubation, the cells in inserts were removed, and the membrane was fixed in 4% paraformaldehyde for 30 min and stained with 0.1% crystal violet for 30 min. Cells were photographed under a light microscope, and the number of migrated cells was counted from five randomly fields of adherent cells.

### Nuclear and cytoplasmic extraction

The cytoplasmic and nuclear fractions were prepared with a kit (Beyotime, Shanghai, China). Briefly, HCT116 and DLD1 cells transfected with si*CUL7* or CUL7 WT were washed twice with PBS, suspended in buffer A, vortexed for 10 s and placed on ice for 15 min. The mixture were then added with buffer B, vortexed for 5 s, placed on ice for 1 min, vortexed for 5 s and centrifuged at 12,000 *g* for 5 min to obtain the cytoplasmic fraction. To obtain the nuclear fraction, a nuclear protein extraction reagent was used to resuspend the pellet obtained from the initial centrifugation. This suspension was incubated on ice for 30 min, vortex for 15 s every 5 min, and centrifuged again at 12,000 for 10 min.

### ROS assay

Cells were transfected with either CUL7 WT and truncation or si*CUL7*. After 36 h, the cells were washed with PBS and incubated with DCF for 30 min at 37 °C. Then, the cells were washed again with PBS and lysed with ROS lysis buffer (150 mM NaCl, 10 mM Tris-base, 0.1 mM EDTA^.^Na_2_, 0.5% Triton X-100) for 10 min. For the animals studies, tissues were lysed with ROS lysis and then incubated with DCF for 30 min at 37 °C. The fluorescence intensity was measured with a fluorescence spectrometer (FlexStation 3, Molecular Devices, Sunnyvale, CA, USA) at 485 nm excitation and 538 nm emission. The fluorescence intensity was standardized to sample with ROS lysis buffer alone, and the cellular ROS level presented as the relative fluorescence intensity per protein concentration.

### Tissue microarray and Immunohistochemistry (IHC)

The tissue microarrays containing 92/80 cases of CRC tissues and 75/80 matched adjacent normal tissues (HColA180Su19/HColA160CS01) were purchase from Shanghai Outdo Biotech, China. The tissue slides were incubated with normal serum to block non-specific binding. Then, the slides were incubated with anti-CUL7 antibody (1:3000/1:500) or anti-KEAP1 antibody (1:500) overnight at 4 °C, followed by addition of a biotinylated secondary antibody and an avidin-biotin peroxidase reagent. After washing with PBS, the slides were visualized by incubating with 3,3-diaminobenzidine (Dako), and counterstained with hematoxylin. For xenograft experiments, tumor slides were incubated with anti-CUL7 (1:50), anti-KEAP1 (1:50), anti-Ki67 (1:600), anti-cleaved Caspase-3 (1:800) or anti-E-cadherin (1:4000). The immunostaining was scored by pathologists in a blinded manner. The stainings were assessed according to the intensity (0: negative, 1: weak, 2: moderate, 3: strong) and the proportion of positive cells (0: 1–5%, 1: 6–25%, 2: 26–50%, 3: 51–75%, 4: 76–100%). The assessment score was the multiply of the intensity and the proportion of positive cells.

### Histology

Mice tumors and colon tissues were carefully isolated and immersed in 4% paraformaldehyde. The tissues were embedded in paraffin and cut into 4 μm sections. The slides were then stained with hematoxylin-eosin (HE) according to the standard histopathological techniques. The mucosal damage score was determined according to a previously described method [21]. The following three parameters were used: surface epithelial loss, crypt destruction and inflammatory cell infiltration into the mucosa. A score of 0–4 was then assigned to each parameter: 0 = no change; 1 = localized and mild; 2 = localized and moderate; 3 = extensive and moderate; and 4 = extensive and severe. The sum of the scores from all three parameters was defined as the mucosal damage score for each animal.

### Statistical analysis

*Data* were reported as mean ± standard deviation (SD). Statistical significances between two groups were determined using an unpaired, two tailed Student’s *t*-test. For multiple group comparison, one-way or two-way ANOVA was used, followed by either a Dunnett or Tukey *post hoc test*. The association between CUL7 expression and patient survival was analyzed using the Kaplan-Meier method, with survival curves compared through log-ran tests. Cox regression, both univariate and multivariate, was used to assess variant by SPSS and a hazard ratio (HR) with 95% confidence interval (CI) is reported. Correlation analysis was analyzed using two-tailed Spearman analysis with 95% CI. GraphPad Prism 8 (GraphPad Software Inc., La Jolla, CA, USA) was used for data analysis. A *p* < 0.05 was considered statistically significant.

## Results

### CUL7 is highly expressed in colon cancer and indicates a poor prognosis

**Fig. 1:**
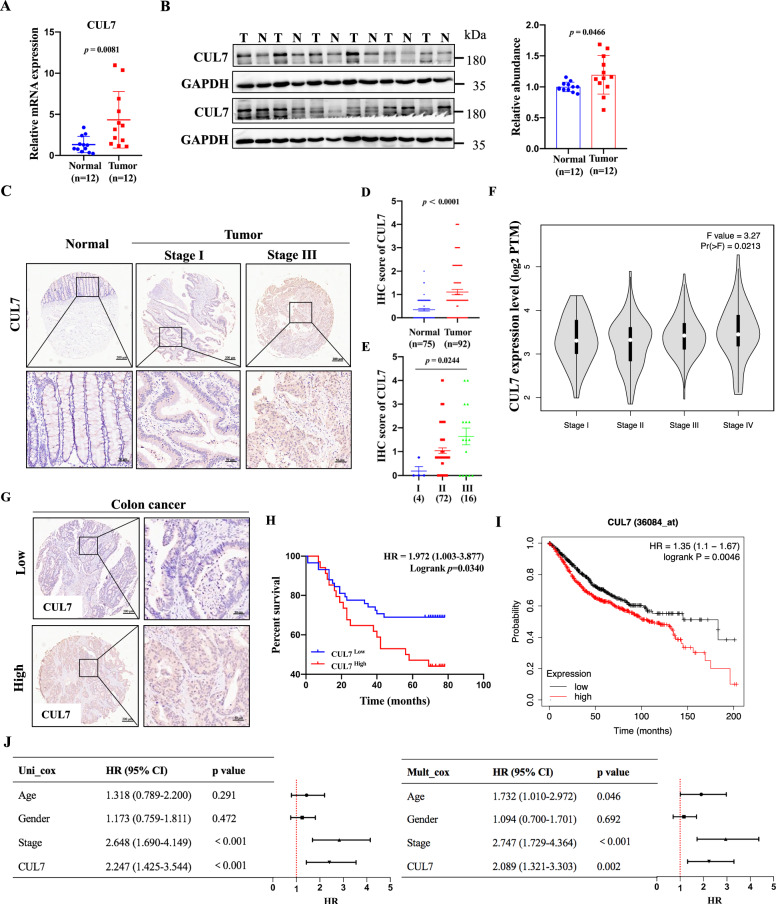
Higher level of CUL7 in colon cancer correlates with poor prognosis. **A** *CUL7* mRNA levels in 12 colon cancer tissues and 12 adjacent normal tissues by qPCR. **B** CUL7 protein expression was detected in 12 colon cancer tissues and 12 adjacent normal tissues by Western blotting and quantification of protein levels. **C**, **D** Representative images of IHC staining and the IHC scores of CUL7 in 92 colon cancer tissues and 75 adjacent normal tissues. Scale bars represent 200 μm. **E** Relative IHC scores of CUL7 across in different tumor grades. **F** Correlation between CUL7 expression and tumor stages of colon cancer was analyzed by GEPIA2 (*n* = 367). **G,** **H** Representative images of IHC and Kaplan-Meier analysis of percent survival based on CUL7 levels in colon cancer patients (low expression of CUL7/high expression of CUL7: *n* = 58/34). Scale bars represent 200 μm. **I** Kaplan-Meier analysis of percent survival of CUL7 in colon cancer patients by using Kaplan-Meier Plotter (low expression of CUL7/high expression of CUL7: *n* = 457/604). **J** Univariate and multivariate cox regression of CUL7 expression for overall survival in colon cancer patients (*n* = 362). Data were analyzed using two-tailed Student’s *t*-test (**A**, **B**, **D**), one-way ANOVA (**E**), two-side log-rank test (**H**, **I**) and cox regression (**J**). Data were represented as mean ± SD.

To evaluate the clinical relevance of CUL7 in colon cancer, we first interrogated *CUL7* mRNA levels in colon cancer and normal colon tissue samples from the First Affiliated Hospital of Xi’an Jiaotong University. Compared with normal colon tissues, CUL7 expression was significantly increased in colon cancer tissues (Fig. 1A). We further validated these findings using The Cancer Genome Atlas (TCGA) cohort via UALCAN. Comparative analysis revealed significantly elevated CUL7 transcript levels in tumor tissues relative to matched adjacent normal tissues (Fig. S1A). Stratified analyses further demonstrated that high *CUL7* mRNA expression was associated with advanced tumor stage and nodal metastasis status, but not with gender or age (Fig. S1B–E).

To validate these findings at the protein level, we detected the expression of CUL7 in colon cancer and normal colon tissues by Western blotting, which showed that CUL7 was markedly upregulated in colon cancer tissues compared with normal colon tissues (Fig. 1B). We then performed IHC analysis using a tissue microarray. Consistent with our transcriptomic data, CUL7 protein expression was markedly higher in tumor tissues than in adjacent normal tissues (Fig. 1C, D and Table S3). Notably, high-grade tumors exhibited significantly higher CUL7 expression than low-grade colon cancer tissues (Fig. 1E and Table S4). Analysis using GEPIA revealed that CUL7 expression was highly correlated with tumor stages progression in colon cancer (Fig. 1F). Moreover, Kaplan-Meier overall survival (OS) analysis indicated that CUL7^High^ patients experienced shorter survival compared with CUL7^Low^ patients (Fig. 1G, H). These results were validated using the Kaplan-Meier Plotter (Fig. 1I). Univariated and multiple cox regression analysis further identified CUL7 expression as an independent factor of OS in colon cancer (Fig. 1J). Collectively, these findings establish CUL7 overexpression plays an important role in colon cancer progression and correlates with poor prognosis.

### CUL7 promotes colon cancer progression in vitro and in vivo

**Fig. 2:**
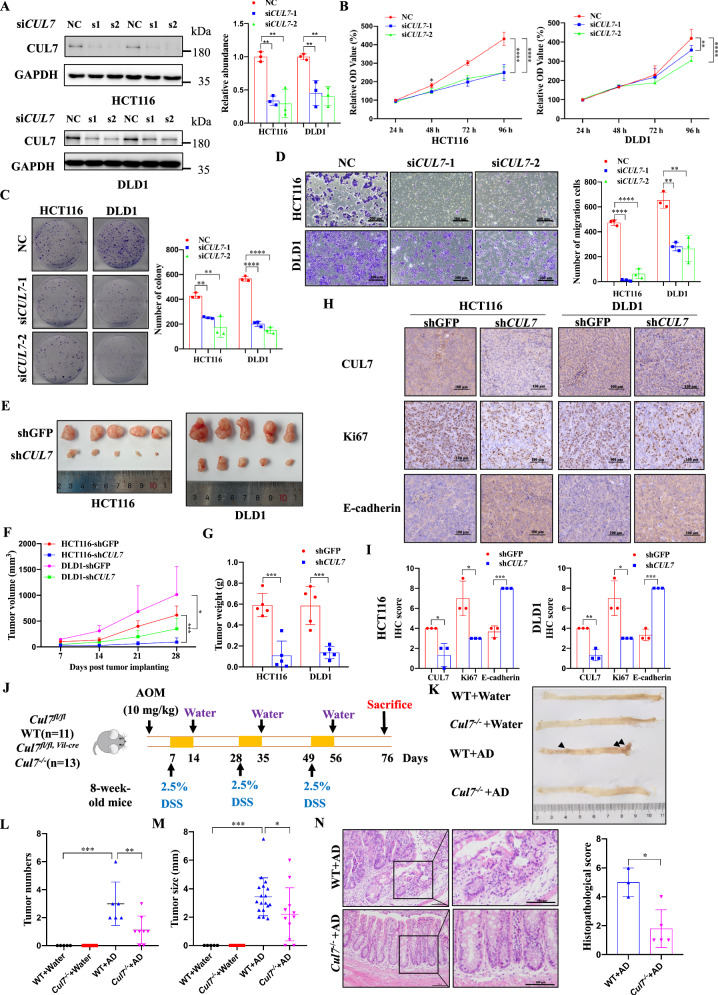
CUL7 promotes colon cancer progression in vitro and in vivo. **A** Western blotting analysis of CUL7 expression in HCT116 and DLD1 cells following *CUL7* knockdown and quantification of protein levels. **B** Effect of *CUL7* deficiency on cell proliferation of HCT116 and DLD1 cells was assessed by MTT assay. **C** Representative images of colony formation in HCT116 and DLD1 cells and quantification of colony number. **D** Representative images of migrated HCT116 and DLD1 cells in migration assay and quantification of migrated cells. Scale bars represent 200 μm. **E**–**G** Representative image of tumors, average tumor volumes over time and weights of tumors at day 28 d for nude mice bearing HCT116 and DLD1 tumors subjects to the indicated treatment. Cells stably expressing the indicated shRNAs (shGFP and sh*CUL7*) were subcutaneously injected into nude mice respectively. *n* = 5 mice per group. **H**, **I** Representative images of IHC staining and the IHC scores of CUL7, Ki67 and E-cadherin in indicated HCT116 and DLD1 tumors of nude mice. Scale bars represent 100 μm. **J** Scheme for AD mouse model. **K** Representative images of colon tissue at sacrifice (WT+Water/*Cul7*^-/-^+Water/WT + AD/*Cul7*^-/-^+AD: *n* = 5/5/6/8). **L**, **M** Tumor number and tumor size in WT and *Cul7* knockout mice subjected to water or AD (WT+Water/*Cul7*^-/-^+Water/WT + AD/*Cul7*^-/-^+AD: *n* = 5/5/6/8). **N** Representative images of HE staining in colon tissue from AD-treated female WT and *Cul7* knockout mice and histopathological score (female WT + AD/ female *Cul7*^-/-^+AD: *n* = 3/5). Scale bars represent 100 μm. *p <0.05, **p ≤ 0.01, ***p ≤ 0.001, ****p ≤ 0.0001. Data were analyzed using one-way ANOVA (**A**, **C**, **D**, **L**, **M**), two-way ANOVA (**B**, **F**), and two-tailed Student’s *t*-test (**G**, **I**, **N**). Data were represented as mean ± SD.

To investigate the oncogenic role of CUL7 in vitro, we investigated its effects on cell proliferation in colon cancer cells. Firstly, we detected the level of CUL7 in different colon cancer cell lines in comparison with the normal colon cell line NCM460. The results showed that HCT116 and DLD1 cells exhibited higher CUL7 expression (Fig. S2A). We then performed RNA interference-mediated knockdown of *CUL7* in HCT116 and DLD1 colon cancer cell lines using two independent siRNA sequences (Fig. 2A). *CUL7* knockdown significantly reduced cell viability (Fig. 2B) and colony formation (Fig. 2C) in both cell lines. Next, to study the role of CUL7 in cell migration, we performed Transwell chamber assays. The number of migrated cells was significantly decreased in the si*CUL7* group compared with the NC group (Fig. 2D). Conversely, CUL7 overexpression in HCT116 and DLD1 cells enhanced cell viability, colony formation capacity and migration in comparison to control cells (Fig. S2B–E). We next established a subcutaneous xenograft model using *CUL7*-knockdown cells and control cells (Fig. S2F). Tumor volume and weight were significantly reduced in the *CUL7* knockdown group compared with controls (Fig. 2E–G). IHC analysis revealed decreased Ki67 level and increased E-cadherin expression in the *CUL7* knockdown tumors, indicating suppressed proliferation and invasive capacity in vivo (Fig. 2H, I).

To further explore the potential association of CUL7 expression with CRC development in vivo, we generated intestine-specific *Cul7* knockout mice (*Cul7*^*fl/fl, Vil-cre*^) and established CRC in these mice by AOM/DSS (AD) (Fig. 2J and Fig. S3A, B). To verify the knockout efficiency, colon tissues from WT and *Cul7*^-/-^ mice were analyzed, showing a significant reduction of CUL7 protein levels by Western blotting (Figure. S3C). Notably, while *Cul7* deficiency did not significant affect body weight, clinical assessment revealed lower scores compared with AD-treatment WT controls (Fig. S3D, E). Importantly, ablation of *Cul7* resulted in a marked reduction in both the tumor number and size in the AD model (Fig. 2K–M). HE staining revealed substantial crypt loss and surface epithelial loss in WT mice treated with AD, alongside the inflammatory cell infiltration into the mucosa and submucosa (Fig. 2N). Taken together, these complementary loss- and gain-of-function experiments demonstrate that CUL7 critically regulates colon cancer progression in vitro and in vivo.

### CUL7 stabilizes and interacts with KEAP1

**Fig. 3:**
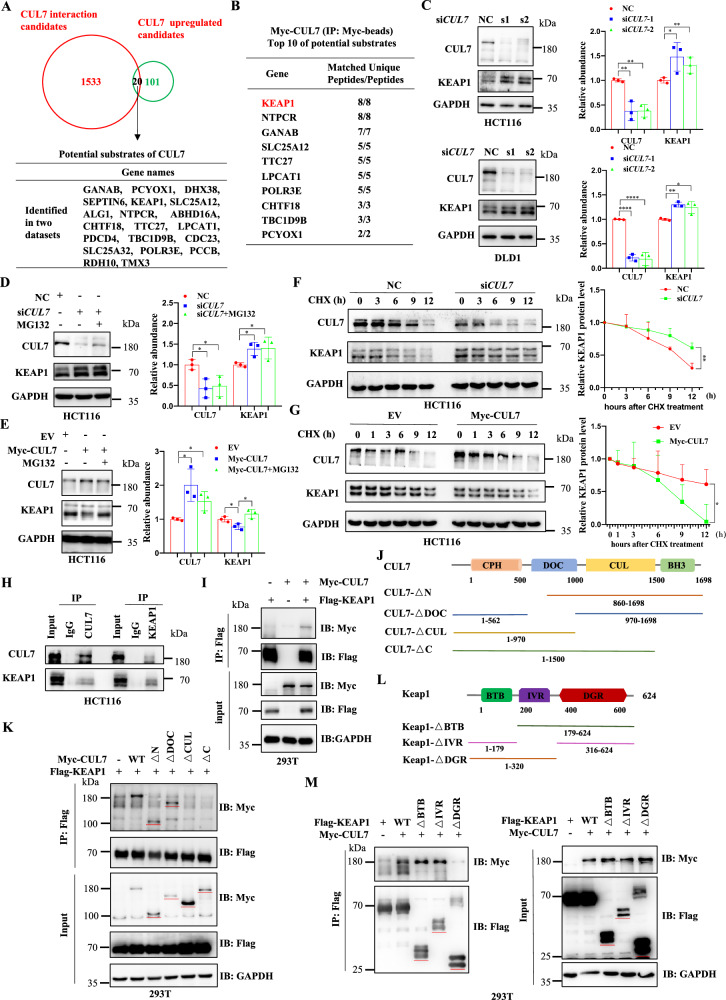
CUL7 modulates KEAP1 stability and interacts with KEAP1. **A** Venn diagram showing the number of CUL7 interaction candidates identified in the CoIP process (red), CUL7 upregulated proteins identified in the quantitative proteomic profiling (green), and overlapping proteins in the datasets. The chart below shows the potential substrates of overlapped proteins. **B** CoIP samples were identified with mass-spectrometry. Top 10 most abundant proteins of the 20 overlapped proteins identified were listed. **C** Western blotting analysis of KEAP1 in HCT116 and DLD1 cells following *CUL7* knockdown and quantification of protein levels. **D** Western blotting analysis of HCT116 cells transfected with NC or si*CUL7*, and treated with 10 μM MG132 for 12 h prior to harvesting and quantification of protein levels. **E** Western blotting analysis of HCT116 cells transfected with EV or Myc-CUL7, and treated with 10 μM MG132 for 12 h before harvesting and quantification of protein levels. **F**, **G** Half-life of KEAP1 in HCT116 cells transfected with si*CUL7* or CUL7 and KEAP1 protein abundance was quantified by Quantity One, which was normalized to GAPDH and then to the t = 0 time point. Cells were treated with 200 μg/mL CHX for the indicated time period prior to harvesting. **H** Endogenous CoIP assay analyzes the interaction of endogenous CUL7 and KEAP1 in HCT116 cells. **I** IB analysis of input and IP derived from 293 T cells transfected with CUL7 and KEAP1 constructs. Cells were treated with 10 μM MG132 for 12 h before harvesting. **J** Schematic of CUL7 domains including CPH, DOC, CUL and BH3 domains. **K** 293 T cells overexpressing full-length CUL7 and various CUL7 truncations (△N, △DOC, △CUL, △C) were immunoprecipitated with indicated antibody. Cells were treated with 10 μM MG132 for 12 h prior to harvesting. Red line marks CUL7 truncation fragment. **L** Schematic of KEAP1 domains including BTB, IVR and DGR domain. **M** 293 T cells overexpressing full-length KEAP1 and various KEAP1 truncations (△BTB, △IVR, △DGR) were immunoprecipitated with indicated antibody. Cells were treated with 10 μM MG132 for 12 h before harvesting. Red lines marks CUL7 and KEAP1 truncation fragment. *p < 0.05, **p ≤ 0.01, ****p ≤ 0.0001. Data were analyzed using one-way ANOVA (**C**, **D**, **E**) and two-way ANOVA (**F**, **G**). Data were represented as mean ± SD.

To elucidate the molecular mechanisms underlying CUL7’s regulation of colon cancer cell survival, we performed CoIP coupled with MS to identify CUL7-interacting proteins, resulting in 1533 candidate interactors. Subsequently, by using quantitative proteomic profiling in HCT116 cells following *CUL7* knockdown (si*CUL7* vs. NC, 48 h treatment), we observed 198 differentially expressed proteins (101 upregulated, fold change [FC] ≥ 2.0, *P* ≤ 0.05; 97 downregulated, FC ≤ 0.5, *P* ≤ 0.05; Fig. S4A). To further identify potential substrates of CUL7, we combined the CoIP dataset and CUL7 upregulated dataset, and obtained 20 overlapping proteins (Fig. 3A). Among the 20 proteins, KEAP1 ranked as a top candidate substrate of CUL7 (Fig. 3B). Western blotting validation confirmed that *CUL7* depletion increased KEAP1 protein levels in both HCT116 and DLD1 cells (Fig. 3C), consistent with proteomic findings.

Analysis via cBioPortal indicated that reduced *KEAP1* expression (4%) and mutation (1%) are prominent characteristics in colon cancer (Fig. S4B). Furthermore, *CUL7* depletion did not affect the mRNA levels of *KEAP1* (Fig. S4C, D), suggesting post-translational regulation of KEAP1 by CUL7. To test whether KEAP1 degradation is proteasome-dependent, *CUL7*-depleted HCT116 cells were treated with the proteasome inhibitor MG132. *CUL7* knockdown increased KEAP1 protein levels, whereas MG132 treatment did not further amplify the differences (Fig. 3D). This observation might be explained by the fact that *CUL7* depletion itself already leads to substantial accumulation of KEAP1. Conversely, CUL7 overexpression resulted in a decrease in KEAP1 levels in HCT116 cells, and this reduced Keap1 protein levels could be restored by MG132 (Fig. 3E). To further establish that CUL7 affects KEAP1 stability per se, we treated the cells with the protein synthesis inhibitor cycloheximide (CHX) and analyzed the half-life of the KEAP1 protein. The half-life of endogenous KEAP1 was found to be extended in cells lacking *CUL7* (Fig. 3F and Fig. S4E), whereas it was shortened in cells overexpressing CUL7 (Fig. 3G). These results collectively demonstrate that CUL7 stabilizes KEAP1 in colon cancer cells.

To further investigate the interaction between CUL7 and KEAP1 in the regulation of KEAP1 stability, we conducted binding assays. Our findings indicated that CUL7 interacted with KEAP1 in two colon cancer cell lines (Fig. 3H and Fig. S5A). Furthermore, co-expression of CUL7 and KEAP1 in 293 T cells revealed that exogenous CUL7 interacted with exogenous KEAP1 (Fig. 3I). Confocal microscopy analysis further substantiated this interaction, showing that the two proteins predominantly colocalized in the cytoplasm, with a co-localization ratio of 0.852 (Manders’ M2) in HCT116 cells (Fig. S5B). Notably, FBXW8, a classical adaptor of CUL7, did not interact with KEAP1 (Fig. S6A, B). To delineate the critical regions involved in the CUL7-KEAP1 interaction, we constructed a series of truncated mutants for both CUL7 and KEAP1. For CUL7, we generated four truncations: △N (lacking the N-terminal CPH domain), △DOC (lacking the DOC domain), △CUL (lacking the CUL and BH3 domains), and △C (lacking the C-terminal BH3 domain) (Fig. 3J). CoIP analysis revealed that the C-terminal region of CUL7 is essential for its interaction with KEAP1 (Fig. 3K). Additionally, we identified the CUL7-binding region of KEAP1 as the DGR domain, as the KEAP1 mutant lacking the C-terminal DGR domain (△DGR) failed to interact with CUL7 in cells (Fig. 3L, M). The CUL family comprises seven proteins, with KEAP1 frequently reported as an adaptor for the CUL3 E3 ligase complex, which ubiquitinates NRF2, thereby inhibiting NRF2 activation and subsequent antioxidant gene expression [15]. To ascertain whether other CUL proteins influence the ubiquitination and degradation of KEAP1, we examined the binding of KEAP1 with additional CUL family proteins and confirmed that CUL3 also interacted with KEAP1 (Fig. S7A), consistent with previous studies. Notably, knockdown of *CUL3* did not affect KEAP1 protein levels in HCT116 and DLD1 colon cancer cells, indicating a distinct regulatory role of CUL7 (Fig. S7B).

### CUL7 ubiquitinates KEAP1 through K29- and K48-linked ubiquitin chains, and its C-terminus is essential for oncogenic function

**Fig. 4:**
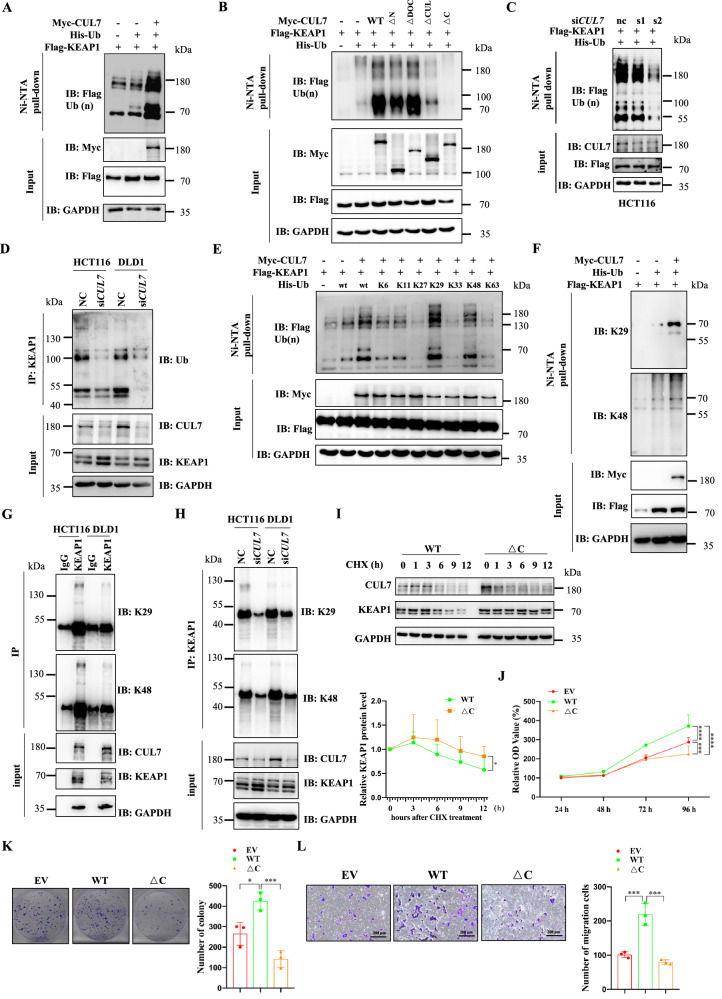
CUL7 ubiquitinates KEAP1 via K29 and K48-linked chains, and C-terminus is essential for its oncogenic function. **A** IB analysis of input and Ni-NTA pull-down products derived from lysates of 293 T cells transfected with CUL7, KEAP1 and Ub. **B** IB analysis of input and Ni-NTA pull-down products derived from lysates of 293 T cells transfected with KEAP1, Ub, CUL7 or its truncation. **C** IB analysis of input and Ni-NTA pull-down products derived from lysates of *CUL7*-depleted HCT116 cells transfected with KEAP1 and Ub. **D** IB analysis of input and anti-KEAP1 IP derived from lysates of *CUL7*-depleted HCT116 and DLD1 cells using the indicated antibodies. **E** IB analysis of input and Ni-NTA pull-down products derived from lysates of 293 T cells transfected with CUL7, KEAP1 and Ub or its mutants. **F** IB analysis of input and Ni-NTA pull-down products derived from lysates of 293 T cells transfected with CUL7, KEAP1 and Ub with indicated antibodies. **G**, **H** IB analysis of input and anti-KEAP1 IP derived from lysates of HCT116 and DLD1 cells and *CUL7*-depleted HCT116 and DLD1 cells using indicated antibodies. For **A**–**H**, cells were treated with 10 μM MG132 for 12 h prior to harvesting. **I** Half-life of KEAP1 in HCT116 cells transfected with WT or △C and KEAP1 protein abundance was quantified by Quantity One, which was normalized to GAPDH and then to the t = 0 time point. Cells were treated with 200 μg/mL CHX for the indicated time period prior to harvesting. **J** Representative MTT assay of cell growth in HCT116 cells transfected with EV, WT or △C. **K** Representative images of colony formation and quantification of colony number in HCT116 cells transfected EV, WT or △C. **L** Representative images of migration and quantification of migrated cells in HCT116 cells transfected with EV, WT or △C. Scale bars represent 200 μm. *p < 0.05, ***p ≤ 0.001, ****p ≤ 0.0001. Data were analyzed using one-way ANOVA (**K,** **L**) and two-way ANOVA (**I,** **J**). Data were represented as mean ± SD.

As CUL7 complex functions as an E3 ubiquitin ligase, we next investigated whether it ubiquitinates KEAP1. Co-transfection of CUL7, KEAP1, and Ub revealed that CUL7 facilitated ubiquitination levels of KEAP1 (Fig. 4A). In contrast, FBXW8 did not enhance KEAP1 ubiquitination (Fig. S6C), and CUL7 truncations △CUL and △C failed to ubiquitinate KEAP1 in vivo (Fig. 4B). Additionally, *CUL7* depletion led to a reduction of KEAP1 ubiquitination in HCT116 cells (Fig. 4C, D).

To elucidate the specific type of ubiquitin chain on KEAP1 that is modulated by CUL7, we transfected cells with constructs expressing CUL7, KEAP1, and ubiquitin K-6/11/27/29/33/48/63 only constructs (keeping only the indicated lysine while mutating the remaining six lysine residues to arginine). CUL7 enhanced K29- and K48-linked ubiquitination of KEAP1 (Fig. 4E). This finding was further validated using K29- and K48-linkage-specific ubiquitin antibodies (Fig. 4F). Moreover, endogenous ubiquitination assay confirmed elevated levels of K29- and K48-linked ubiquitination of KEAP1 in both colon cancer cells (Fig. 4G), which was reduced upon *CUL7* depletion (Fig. 4H). Taken together, these results demonstrate that CUL7 regulates KEAP1 abundance primarily through K29- and K48-linked ubiquitination.

The above findings indicated that CUL7 interacts with and ubiquitinates KEAP1 via its C-terminal domain (amino acid (aa) 1501-1698). Consequently, we hypothesized that the C-terminus of CUL7 is crucial for its functional activity. Functional assays showed that CUL7 △C failed to shorten the half-life of KEAP1 (Fig. 4I). In contrast to EV, exogenous overexpression of CUL7 wild type (WT), rather than CUL7 △C, dramatically enhanced cell proliferation, colony formation and cell migration in HCT116 cells (Fig. 4J-L). Overall, these data indicate C-terminus of CUL7 is a critical region for preserving its biological functions.

### CUL7 promotes cell growth of colon cancer via NRF2 signaling

**Fig. 5:**
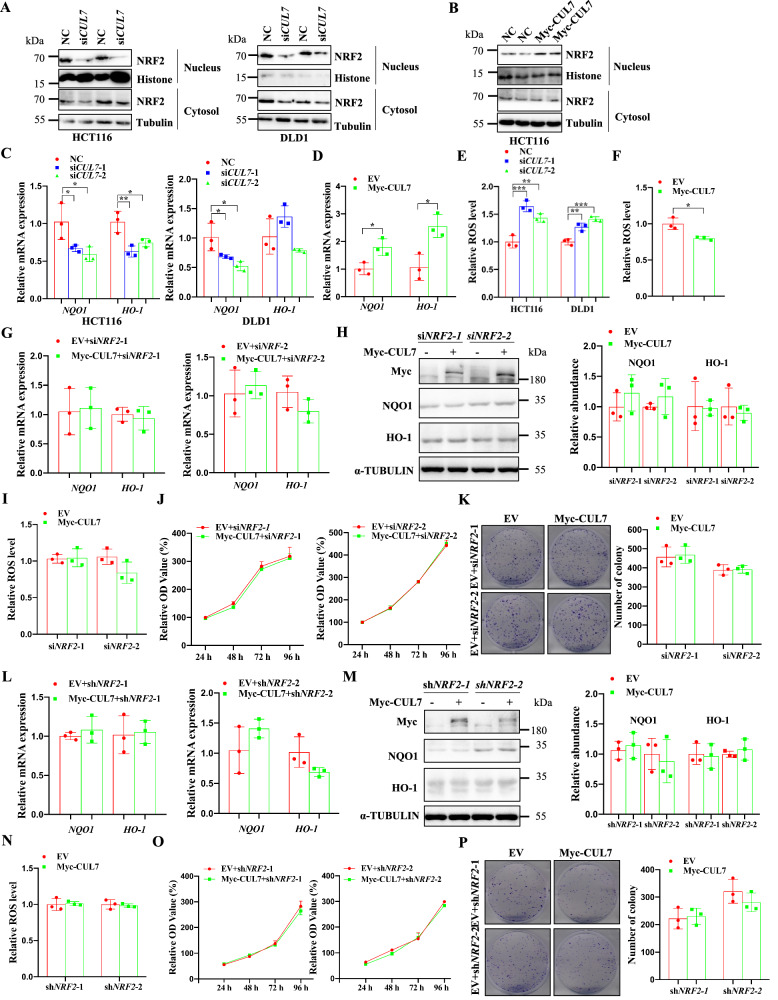
CUL7 promotes cell proliferation of colon cancer via NRF2 signaling. **A** Nuclear/cytosolic fractionation assay and western blotting analysis of NRF2 in HCT116 and DLD1 cells transfected with NC or si*CUL7*. **B** The level of NRF2 in nuclear and cytosolic fractions of EV or Myc-CUL7-expressing HCT116 cells. **C**, **D** Relative mRNA fold changes of antioxidant genes, including *NQO1* and *HO-1* in *CUL7*-depleted and CUL7-expressing colon cancer cells. **E**, **F** Relative ROS levels in *CUL7*-depleted and CUL7-expressing colon cancer cells. **G** Relative mRNA fold change of antioxidant genes, including *NQO1* and *HO-1* in HCT116 cells. **H** Western blotting analysis of NQO1, HO-1 and Myc-tag in HCT116 cells and quantification of protein levels. **I** Relative ROS levels in HCT116 cells. **J** Representative MTT assay of cell growth in HCT116 cells. **K** Representative images of colony formation and quantification of colony number in HCT116 cells. For **G**–**K**, cells were expressed EV or Myc-CUL7 with concurrent *NRF2* knockdown. **L** Relative mRNA fold change of antioxidant genes, including *NQO1* and *HO-1* in stable *NRF2*-depleted HCT116 cells. **M** Western blotting analysis of NQO1, HO-1 and Myc-tag in stable *NRF2*-depleted HCT116 cells and quantification of protein levels**. N** Relative ROS levels in stable *NRF2*-depleted HCT116 cells. **O** Representative MTT assay of cell growth in stable *NRF2*-depleted HCT116 cells. **P** Representative images of colony formation and quantification of colony number in stable *NRF2*-depleted HCT116 cells. For **L**–**P**, stable *NRF2*-depleted cells were expressing EV or Myc-CUL7. *p < 0.05, **p ≤ 0.01, ***p ≤ 0.001. Data were analyzed using one-way ANOVA (**C**, **E**), two-tailed Student’s *t*-test (**D**, **F**, **G**–**I**, **K,** **L**–**N**, **P**) and two-way ANOVA (**J**, **O**). Data were represented as mean ± SD.

As KEAP1 is a well-known inhibitor of NRF2 [22]. We proceeded to examine the role of CUL7 in the modulation of NRF2 activation and the subsequent antioxidant response. We found that the protein levels of NRF2 in both nuclear and cytoplasmic extracts were reduced in HCT116 and DLD1 cells with *CUL7* knockdown (Fig. 5A). Conversely, CUL7 promoted NRF2 accumulation in the nucleus, with no corresponding effect observed in the cytoplasm of HCT116 cells (Fig. 5B). Consistence with this activation, the expression levels of the antioxidant genes HO-1 and NQO1, which are direct targets of NRF2, were markedly diminished upon *CUL7* depletion in HCT116 cells (Fig. 5C). In contrast, CUL7 overexpression significantly enhanced the expression of these antioxidant genes (Fig. 5D). Moreover, ROS levels were notably elevated in HCT116 and DLD1 cells following *CUL7* loss (Fig. 5E), whereas the upregulation of CUL7 resulted in a notable decline in ROS levels in HCT116 cells (Fig. 5F).

Having established that CUL7 regulates cell growth and activates NRF2 signaling, it is imperative to explore the functional interplay between CUL7 and NRF2 in cellular proliferation. We generated *NRF2*-depleted HCT116 cell lines with si*NRF2*, co-expressing either EV or CUL7 (Fig. S8A). *NRF2* depletion nearly abolished CUL7-mediated upregulation of antioxidant genes expression and suppression of ROS production (Fig. 5G–I). Consistent with these findings, the proliferative effect of CUL7 was significantly impaired in *NRF2*-deficient cells (Fig. 5J, K). Additionally, we generated stable *NRF2*-depleted HCT116 and DLD1 cells using sh*NRF2* (Fig. S8B, C). Consistent with siRNA results, stable depletion of *NRF2* inhibited the effect of CUL7 on increasing the levels of antioxidant genes and cell proliferation, while suppressing cellular ROS (Fig. 5L–P and Fig S8D–H). Altogether, these results demonstrate that the role of CUL7 in promoting cell proliferation is dependent on the NRF2 signaling pathway.

### CUL7 promoted colon cancer progression in vitro and in vivo through KEAP1

**Fig. 6:**
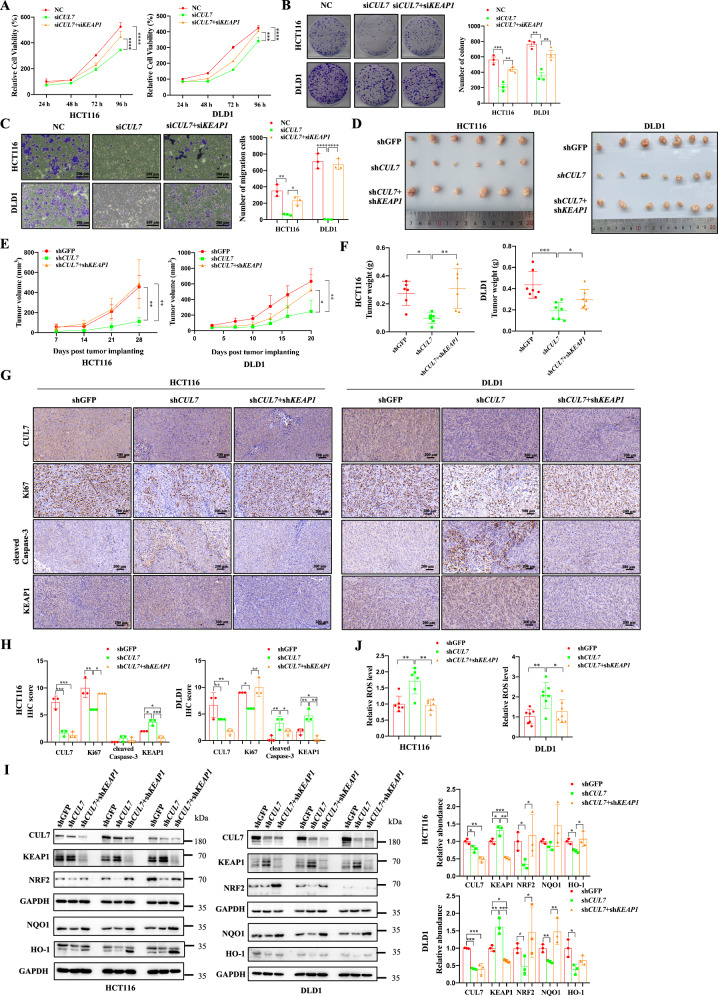
CUL7 promotes colon cancer promotion through KEAP1 in vitro and in vivo. **A** MTT assay showed the proliferative capabilities in HCT116 and DLD1 cells. **B** Representative images of colony formation in HCT116 and DLD1 cells and quantification of colony number. **C** Representative images of migrated HCT116 and DLD1 cells and quantification of migrated cells. Scale bars represent 200 μm. For **A**–**C**, cells were simultaneously transfected with si*CUL7* and si*KEAP1*. **D**–**F** Representative image of tumors, average tumor volumes over time and weights of tumors at day 28 d or 20 d for nude mice bearing HCT116 and DLD1 tumors subjects to the indicated treatments. Cells stably expressing the indicated shRNAs (shGFP, sh*CUL7* and sh*CUL7*+sh*KEAP1*) were subcutaneously injected into nude mice respectively. *n* = 6 mice per group (HCT116) or *n* = 7 mice per group (DLD1). **G**, **H** Representative images of IHC staining and the IHC scores of CUL7, Ki67, cleaved Caspase-3 and KEAP1 in indicated HCT116 and DLD1 tumors of nude mice. Scale bars represent 200 μm. **I** Western blotting analysis of CUL7, KEAP1, NRF2, NQO1 and HO-1 in the indicated HCT116 and DLD1 tumors of nude mice and quantification of protein levels. **J** Relative ROS levels in indicated HCT116 and DLD1 tumors of nude mice. *p < 0.05, **p ≤ 0.01, ***p ≤ 0.001, ****p ≤ 0.0001. Data were analyzed using two-way ANOVA (**A,** **E**) and one-way ANOVA (**B**, **C**, **F**, **H**, **I**, **J**). Data were represented as mean ± SD.

Given that CUL7 controls colon cancer cell survival, KEAP1 expression, and NRF2 signaling pathway, it is crucial to explore the role of KEAP1 in mediating the biological activity of CUL7. To test this hypothesis, we simultaneously transfected HCT116 and DLD1 cells with si*CUL7* and si*Keap1* (Fig. S9A). Depletion of *Keap1* alleviated the suppression of cell viability (Fig. 6A), colony formation capacity (Fig. 6B), and cell migration (Fig. 6C) induced by *CUL7* knockdown in both cell lines. We next generated stable HCT116 and DLD1 cell lines depleted of *CUL7* with or without *KEAP1* (Fig. S9B), and subsequently implanted these cell lines into nude mice. The downregulation of *CUL7* reduced tumor size in the nude mice, however, the ablation of *KEAP1* counteracted this decrease in tumor size observed in the sh*CUL7* knockdown cells (Fig. 6D, E). Furthermore, mice with *CUL7* knockdown exhibited a lower tumor weight compared to controls, but the depletion of *KEAP1* mitigated the phenotypic effects associated with *CUL7* knockdown (Fig. 6F). HE staining of tumors in the three groups revealed that tumors derived from mice implanted with sh*CUL7* cells exhibited smaller nuclei and more defined borders, while the downregulation of *KEAP1* hindered the alteration of nuclei and tumor borders in *CUL7*-depleted mice (Fig. S9C). As expected, the depletion of *CUL7* led to an upregulation of KEAP1 and cleaved Caspase-3 levels, and a downregulation of Ki67 levels in the tumors of the mice. The loss of *KEAP1* resulted in decreased expression levels of KEAP1 and cleaved Caspase-3, and an increase in Ki67 expression in *CUL7*-depleted tumors (Fig. 6G–I). Additionally, NRF2 and its downstream antioxidant genes, *NQO1* and *HO-1*, were dramatically reduced, whereas reactive oxygen species (ROS) levels were elevated in tumor xenografts derived from *CUL7*-depleted cells. Conversely, *KEAP1* knockdown significantly recovered the decline in these antioxidant genes and the increase in ROS levels in the tumor xenografts with loss of *CUL7* (Fig. 6I, J). These results suggest that the CUL7 promotes colon cancer progression in vitro and in vivo through KEAP1.

### CUL7 correlates with KEAP1 and NRF2 in human colon cancer specimens

**Fig. 7:**
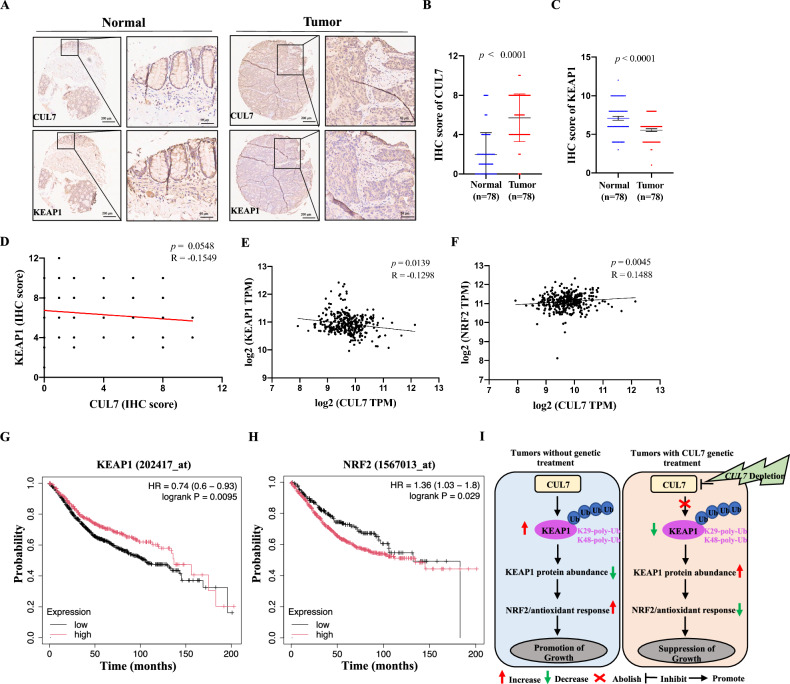
CUL7 correlates with KEAP1 and NRF2 in human colon cancer specimens. **A**–**C** Representative images of IHC staining and the relative IHC scores of CUL7 and KEAP1 in colon cancer tissues (*n* = 78) and adjacent normal tissues (*n* = 78). Scale bars represent 200 μm. **D** Regression analysis comparing CUL7 and KEAP1 protein levels in colon cancer samples (*n* = 152). **E**, **F** Regression analysis comparing CUL7 and KEAP1 or NRF2 mRNA in colon cancer samples from TCGA (*n* = 362). **G**, **H** Kaplan-Meier analysis of percent survival of KEAP1 and NRF2 in colon cancer patients (low expression of KEAP1/high expression of KEAP1: *n* = 719/342; low expression of NRF2/high expression of NRF2: *n* = 231/583). **I** A schematic illustration of the proposed model of mechanistically how CUL7 could regulate the stability of KEAP1, thereby activating NRF2 signaling, promoting colon cancer progression (left panel). However, inhibition of *CUL7* by genetic treatment upregulates KEAP1, setting up a oxidation microenvironment to suppress tumor growth (right panel). Data were analyzed using Student’s *t*-tests (**B**, **C**), Spearman correlation method (**D**–**F**) and two-side log-rank test (**G**, **H**). Data were represented as mean ± SD.

To clarify the clinical association between CUL7 and KEAP1 in colon cancer, we initially examined the levels of KEAP1 in colon cancer tumor tissues and adjacent normal tissues. IHC analysis of tissue microarrays indicated that the majority of tumor tissues exhibited higher CUL7 expression alongside lower KEAP1 expression when compared to adjacent normal tissues (Fig. 7A–C). Correlation analysis further revealed a negatively correlated tendency between CUL7 and KEAP1 protein in the context of colon cancer (Fig. 7D). Consistently, an analysis of data from TCGA database further revealed a significantly negative correlation between CUL7 and KEAP1 mRNA, while an obviously positive correlation between CUL7 and NRF2 mRNA in colon cancer (Fig. 7E, F). Moreover, GEPIA2 analysis showed that NQO1 expression was significantly increased in colon cancer tissues compared to the normal tissues (Fig. S9A), and that CUL7 expression positively correlated with NQO1 levels (Fig. S9B). Additionally, Kaplan-Meier Plotter analysis indicated that KEAP1^Low^, NRF2^High^ or NQO1^High^ patients experienced shorter survival compared to the KEAP1^High^, NRF2^Low^ or NQO1^Low^ patients (Fig. 7G, H and Fig. S9C). Altogether, these results suggest that CUL7 promotes CRC progression, which is mediating by KEAP1-NRF2 signaling pathway in clinical CRC specimens.

## Discussion

Our findings demonstrate that CUL7 mediating KEAP1 ubiquitination, thereby orchestrating NRF2 signaling and promoting colon cancer progression. These mechanistic insights not only advance our understanding of redox regulation in cancer biology but also provide a molecular framework for developing targeted therapeutic strategies for CRC (Fig. 7I).

CRLs, the largest family of ubiquitin ligases, utilize CUL proteins as structural scaffolds for complex assembly. CRLs regulate diverse cellular signaling pathways and have been implicated in the pathogenesis of various human diseases, particularly malignancies [23]. Among CUL family member, CUL7 has emerged as a critical molecule that promotes cancer progression across multiple cancer types, including hepatocellular carcinoma [24–26], breast cancer [27–29], epithelial ovarian cancer [30], esophageal cancer [31], lung cancer [8, 32], pancreatic cancer [33], cervical cancer [29], glioma [34], and colon cancer [12, 13]. However, previous research has primarily focused on correlating CUL7 expression levels with proliferative indices, leaving its comprehensive biological functions, clinical relevance, and molecular mechanisms largely unexplored. In this study, we elucidated the oncogenic role and molecular mechanisms of CUL7 in colon cancer. Our analysis revealed significant upregulation of CUL7 at both transcriptional and translational levels in primary tumor tissues compared to matched adjacent tissues, and high-grade tumors exhibited significantly higher CUL7 expression than low-grade colon cancer tissues. Moreover, *Cul7* loss significantly suppressed tumor growth in AD-induced CRC mice. Complementary in vitro studies demonstrated that *CUL7* depletion obviously impaired cell proliferation and migration of colon cancer, whereas its overexpression conferred a survival advantage. These findings collectively establish CUL7 has a potential oncogenic property in CRC, consistent with its tumor-promoting functions in other malignancies.

CRLs frequently assemble into multimeric complexes to expand substrate diversity and enhance ubiquitination specificity [35, 36]. To date, only 14 substrates have been identified as ubiquitinated targets of CUL7, including CyclinD1 [37], IRS-1 [38], GRASP65 [39], TBC1D3 [40], MRFAP1 [41], HPK1 [33], AID [9], H2B [42], Caspase8 [29], Eag1 [43], MST1 [44], LL5β [45], TET2 [8], ATGL [46]. This limited substrate underscores the critical need to identify novel CUL7-associated targets. Here, we observed a significant accumulation of KEAP1-a key regulator of cancer-associated redox signaling, in *CUL7*-depleted cells. Biochemical analyses revealed that the C-terminal domain of CUL7 interacts with KEAP1 and mediates its ubiquitination. Notably, we demonstrated that CUL7 preferentially catalyzes K29- and K48-linked polyubiquitination of KEAP1. However, the molecular basis underlying this linkage specificity remains unclear. Below is our preliminary analysis based on existing literature and our data. First, as a member of the CRL family, CUL7’s ubiquitination activity depends on recruited E2 enzymes, which have intrinsic preferences for specific ubiquitin linkages (e.g., UBE2R1/2 for K48) [47]. Second, substrate adaptors of CRL complexes may regulate linkage specificity through steric hindrance or ubiquitin-chain elongation interfaces [48]. We found FBXW8 might not be CUL7 adaptor in Keap1 ubiquitination. Therefore, we should further explore CUL7 adaptor to discuss linkage specificity from structural features of the ligase complex. Third, substrates might induce conformational rearrangements in the E2-E3 catalytic center upon binding to E3 complex, thereby selecting specific ubiquitination sites [49]. This mechanism may be analogous to the K48-linked polyubiquitination of KEAP1. The observed K29/K48 chain specificity mediated by CUL7 is likely governed by the combined effects of E2 enzyme selectivity, structural features of the ligase complex, and substrate positioning. Future investigations should employ integrated biochemical, structural, and genetic approaches to systematically explore the molecular underpinnings of this mechanism. Elucidating these principles will advance our understanding of CRL-mediated ubiquitination and provide novel therapeutic strategies for targeting the CRL.

KEAP1, encoded by the *KEAP1* gene, is known to repress the transcriptional activity of NRF2, with the KEAP1/NRF2 axis playing a central role in cellular defense and survival pathway [22]. Loss-of-function mutations in *KEAP1*, which lead to NRF2 activation, are frequently observed in multiple malignancies, including colon cancer, lung cancer, bladder cancer, and breast cancer [50–53]. Consistent with its tumor-suppressive role, *KEAP1* deficiency has been shown to accelerate *Kras*-driven lung cancer [54], enhance survival of hydrogen peroxide-treated hepatocytes [55], and promote colon cancer cell proliferation [56]. Our findings align with these observations, as *KEAP1* depletion robustly enhanced cell survival in colon cancer cells depleted of *CUL7*. However, additional studies have identified that KEAP1 also exhibits a context-dependent role in cancer [57]. KEAP1 undergoes ubiquitination [16] and can be deubiquitinated [58], and further be degraded via autophagy [18, 59] or proteasome-dependent degradation mechanisms [17, 19]. Our results corroborate these findings, establishing that CUL7 promotes ubiquitination and degradation of KEAP1, thereby expanding the mechanistic understanding of KEAP1 regulation in cancer. In accordance with the KEAP1 findings, studies found that NRF2 target gene NQO1 was overexpressed in cancers, high NRF2 levels were associated with poor prognosis in CRC patients [60]. Although NRF2 was originally identified as a protective transcription factor in preventing cancer and other health problems, increasing evidence indicates that aberrant NRF2 activation promotes carcinogenesis, metastasis, and therapeutic resistance [61]. Previous studies have also observed that Nrf2 might promote AD-induced CRC progression [62–65]. In line with these findings, our results demonstrate that CUL7 promotes cell growth of colon cancer in an NRF2-dependent manner. Collectively, our CUL7-KEAP1-NRF2 findings maybe a crucial signaling axis in colon cancer.

## Conclusion

Our study demonstrated that CUL7 catalyzes K29- and K48-linked polyubiquitination of KEAP1, thereby modulating its protein stability. This regulatory mechanism influences NRF2 signaling pathway and promotes colon cancer progression. These findings establish CUL7-KEAP1-NRF2 signaling axis as a key molecular node in colon cancer and highlight its therapeutic potential as a druggable target for colon cancer treatment. In the future study, the use of MS will clarify the ubiquitination sites of KEAP1 by CUL7, providing more mechanism insights into CUL7-mediated ubiquitination of KEAP1. Moreover, the efficacy of CUL7-specific small-molecule inhibitors remains to be validated. Further studies employing these inhibitors to complement the current findings could enhance their translational potential.

## Supplementary information

Full length western blots

Figure S1-S10

Table S1-S4

aj-checklist

## Data Availability

The mRNA expression data and related clinical information (Fig. 1F and Figure S1A-E, 10A) for the samples were downloaded from UALCAN (http://ualcan.path.uab.edu) and GEPIA2 (http://gepia2.cancer-pku.cn/#index). The survival curve (Figs. 1I, 7G, 7H and Figure S10C) were got from Kaplan–Meier Plotter (https://kmplot.com/analysis/). The correlation data (Fig. S10B) was obtained from GEPIA2. The remaining data are available within the Article, Supplementary Information or Original Data file.
