# Supplementary material for: CUL7-mediated KEAP1 ubiquitination promotes the progression of colon cancer via NRF2 signaling
Source: Cell Death Dis. 2026 Jun 8;17(1):712. doi: 10.1038/s41419-026-08929-1 (PMC13462133; doi:10.1038/s41419-026-08929-1)

Western blotting images related to Figure 1.

A

Fig. 1B

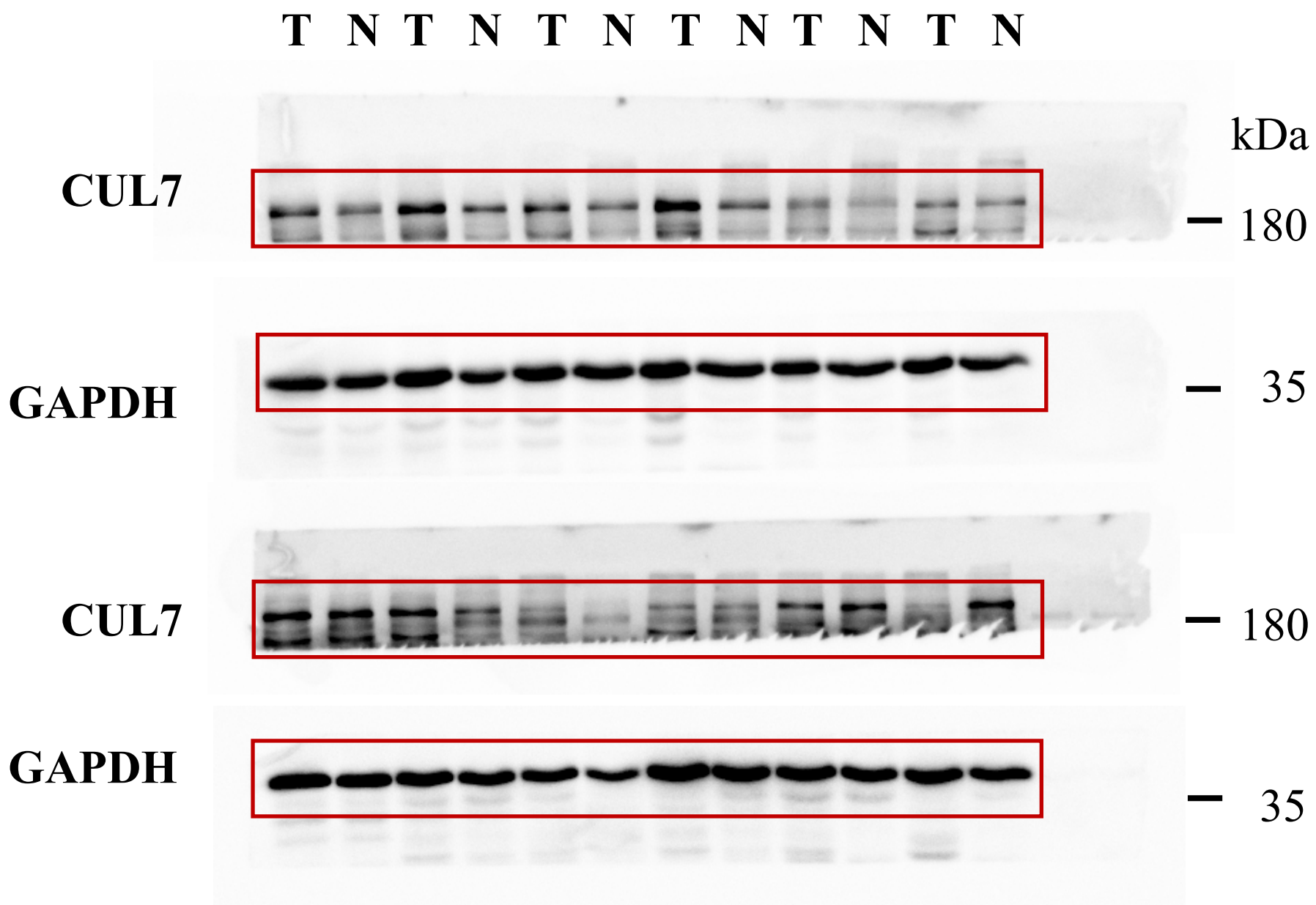

Western blotting images related to Figure 2.

A

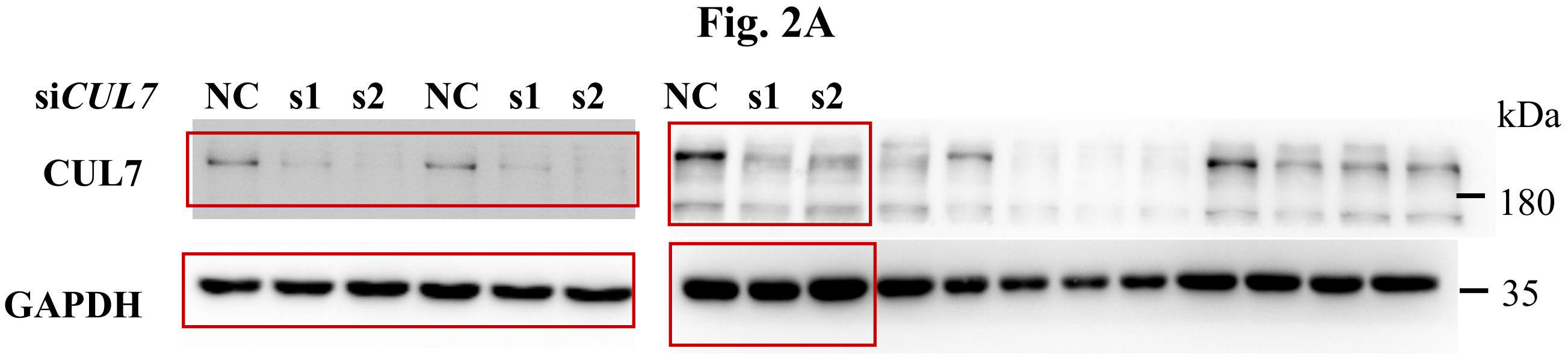

HCT116

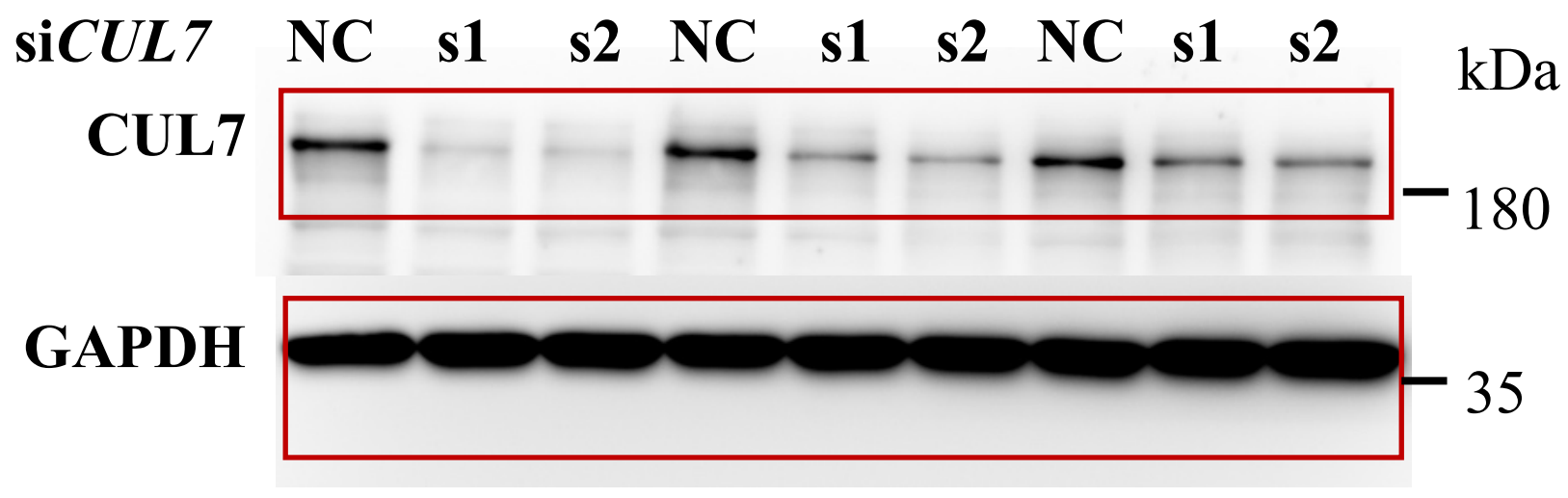

DLD1

Western blotting images related to Figure 3.

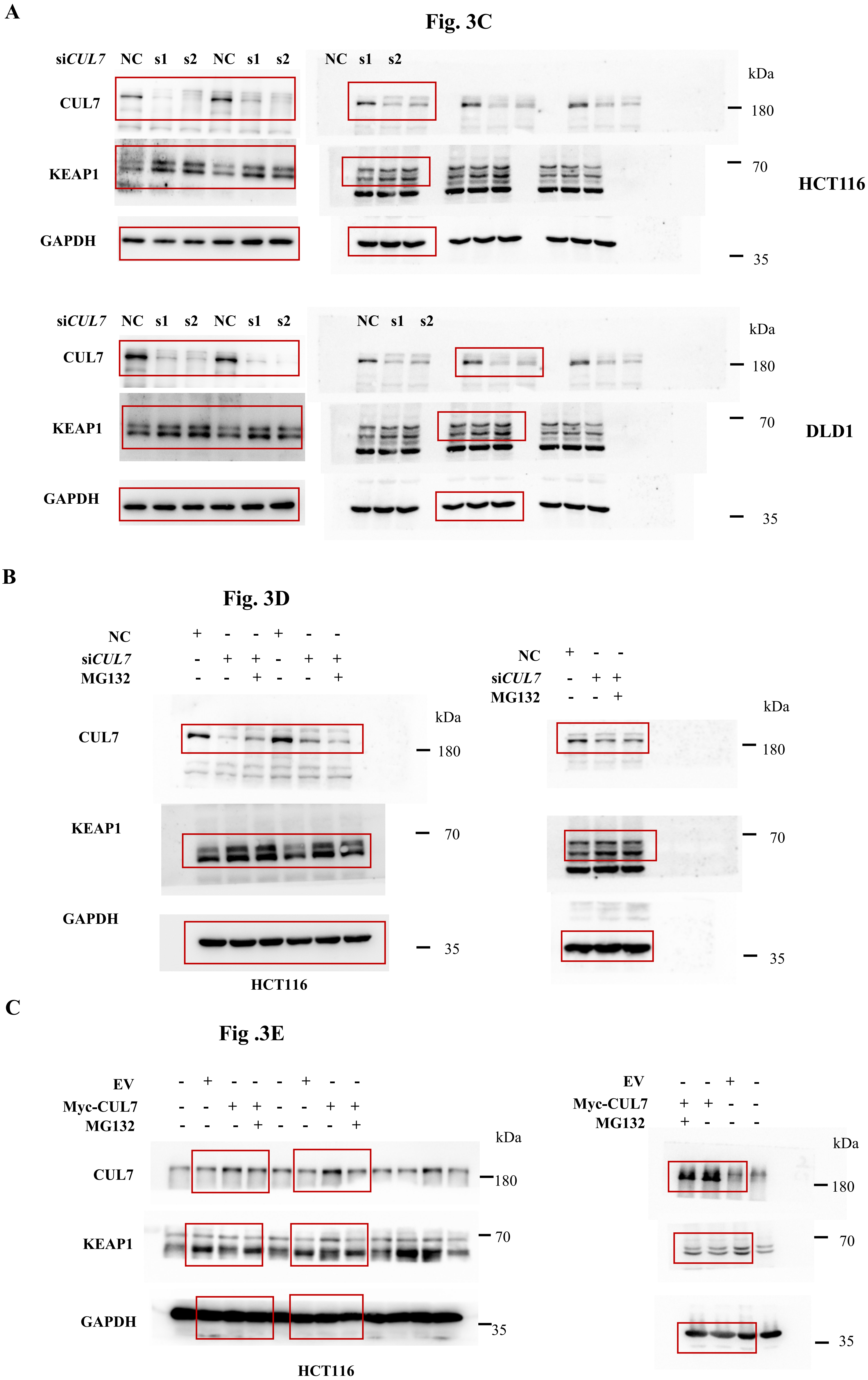

Western blotting images related to Figure 3.

D

Fig. 3F

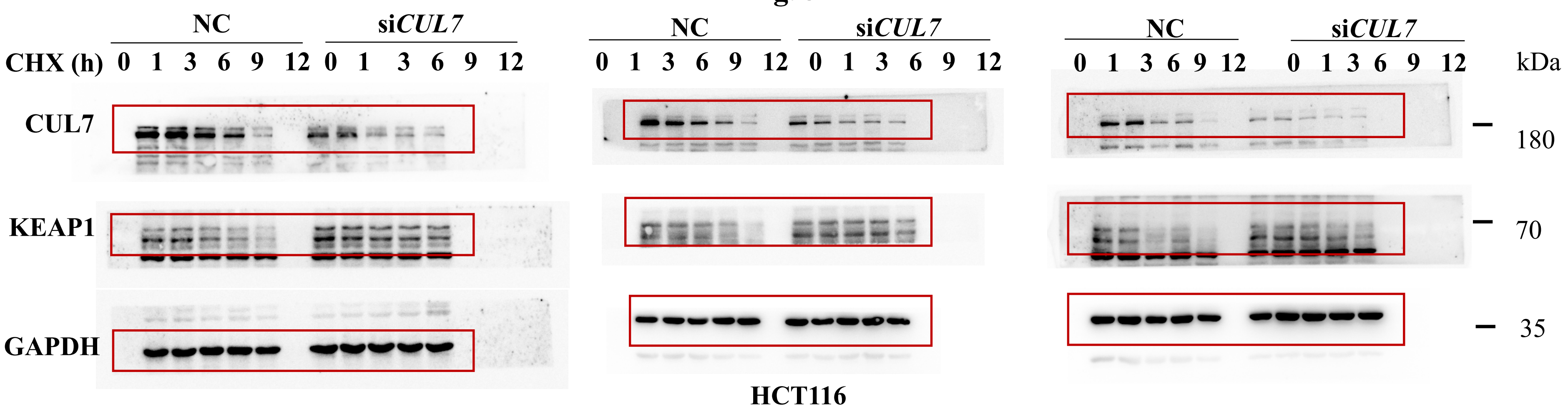

E

Fig. 3G

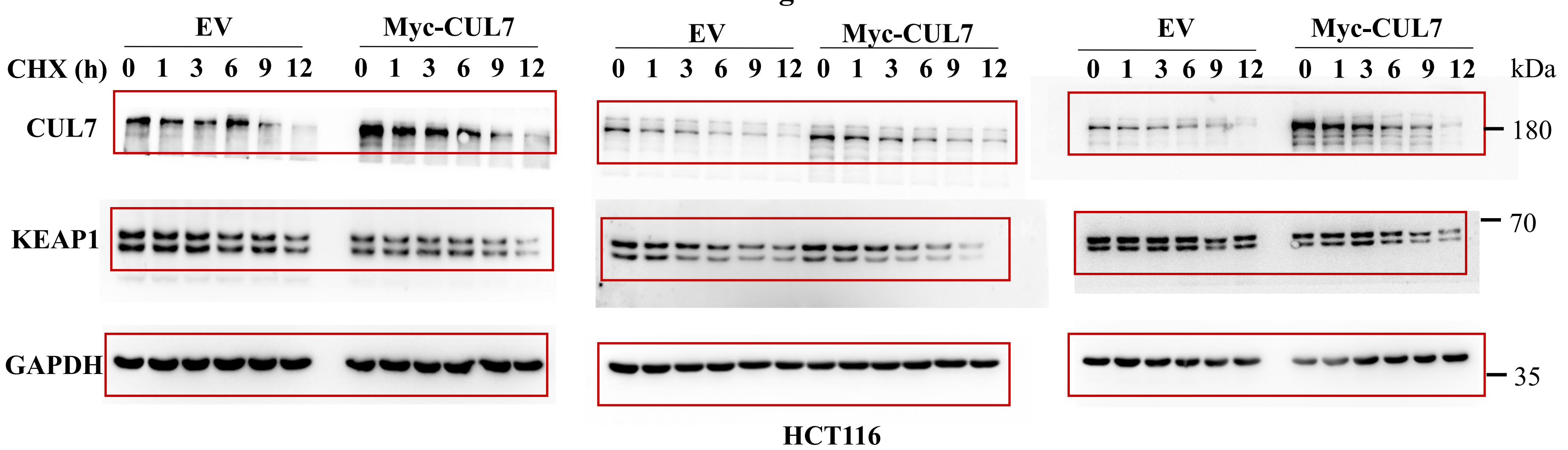

F

Fig. 3H

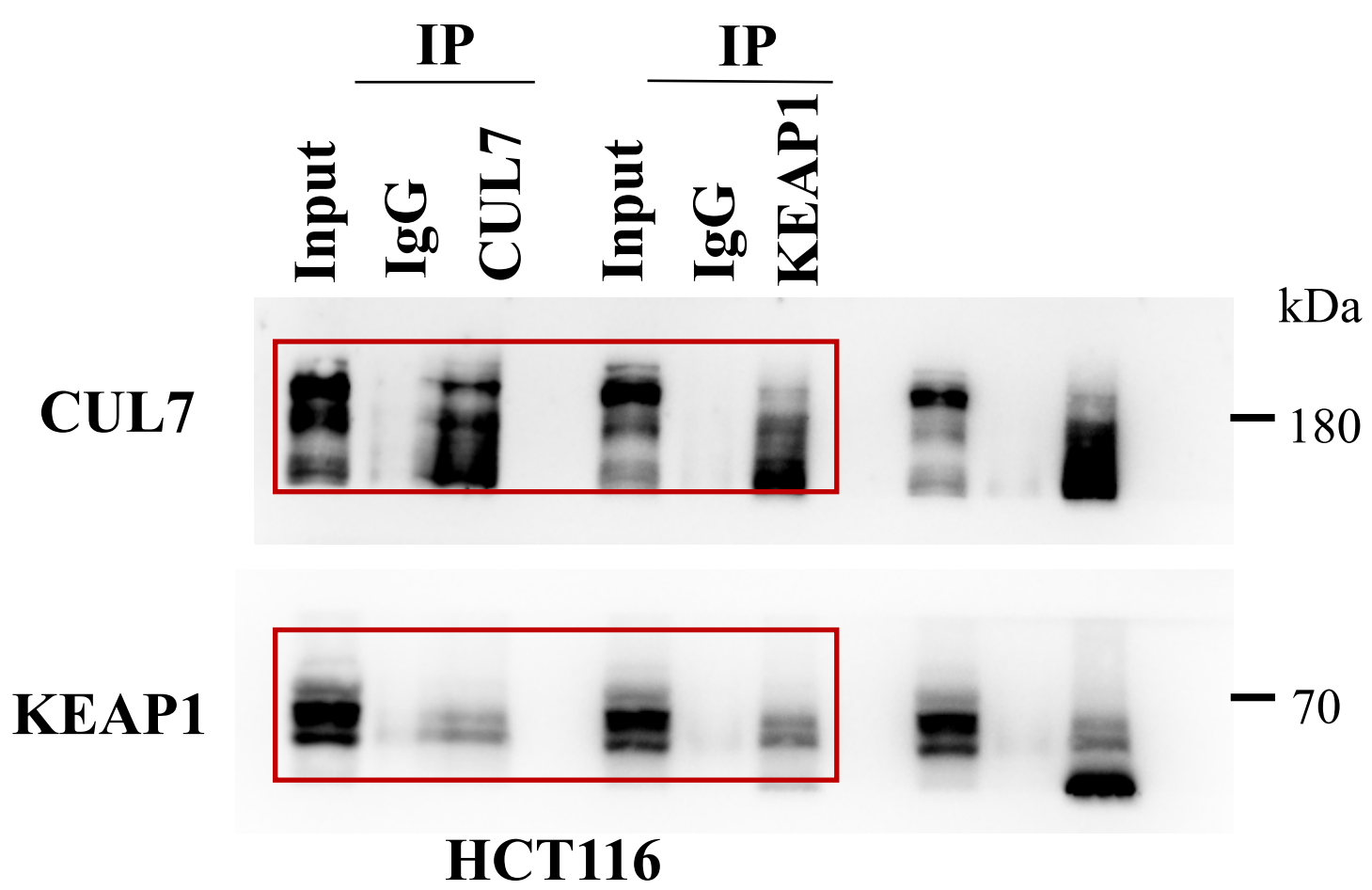

Western blotting images related to Figure 3.

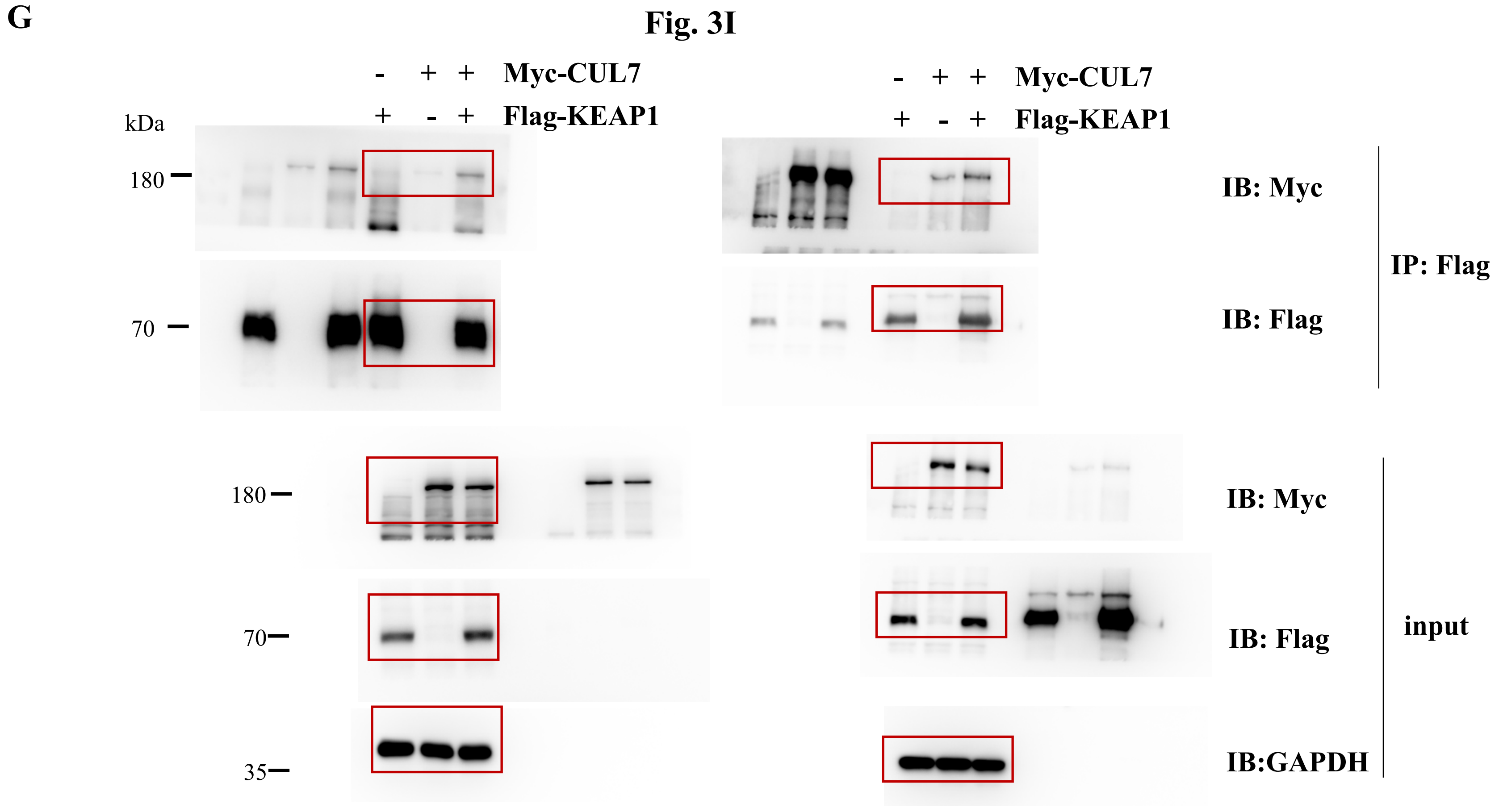

Western blotting images related to Figure 3.

H

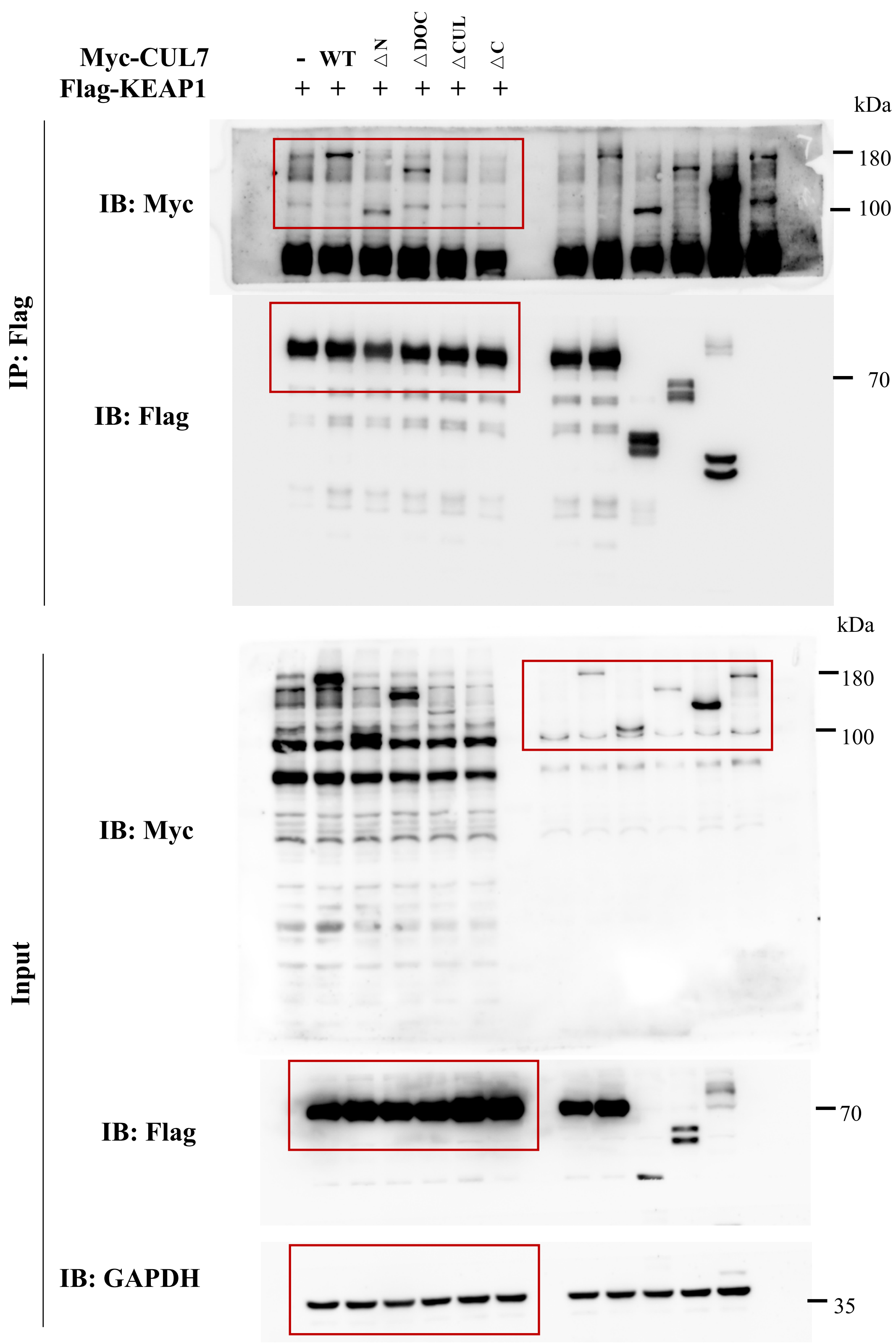

Fig. 3K

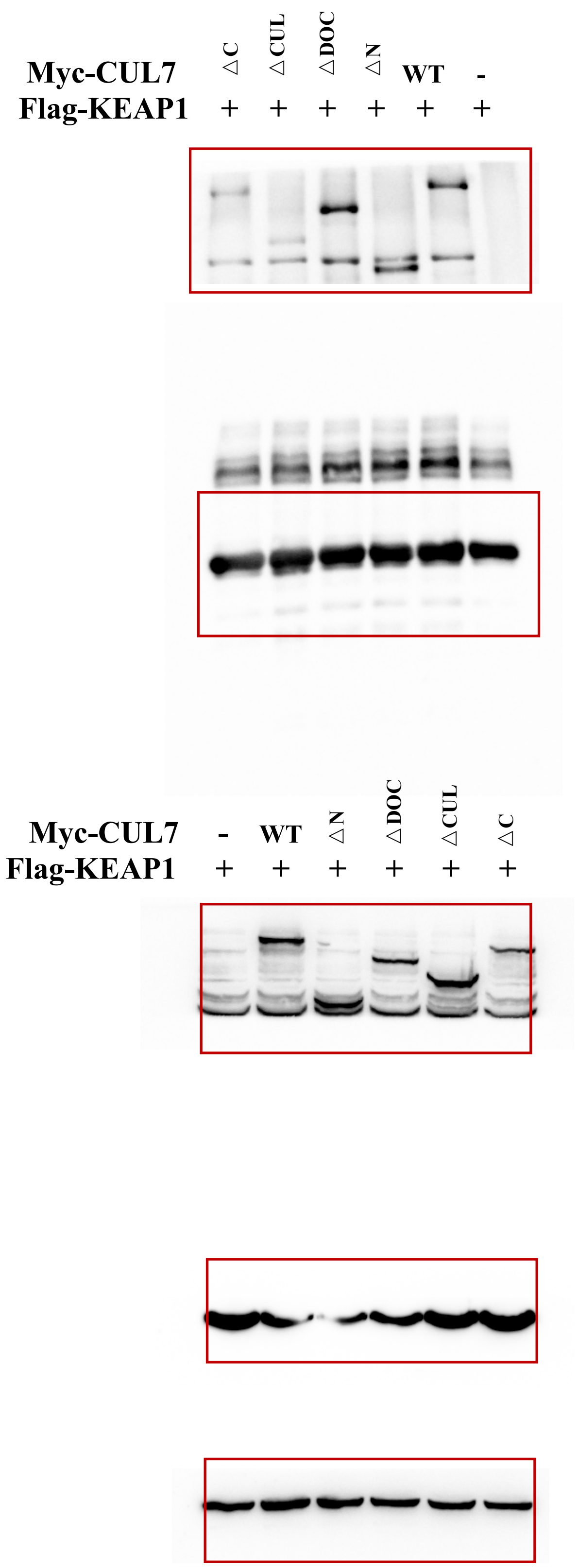

Western blotting images related to Figure 3.

I

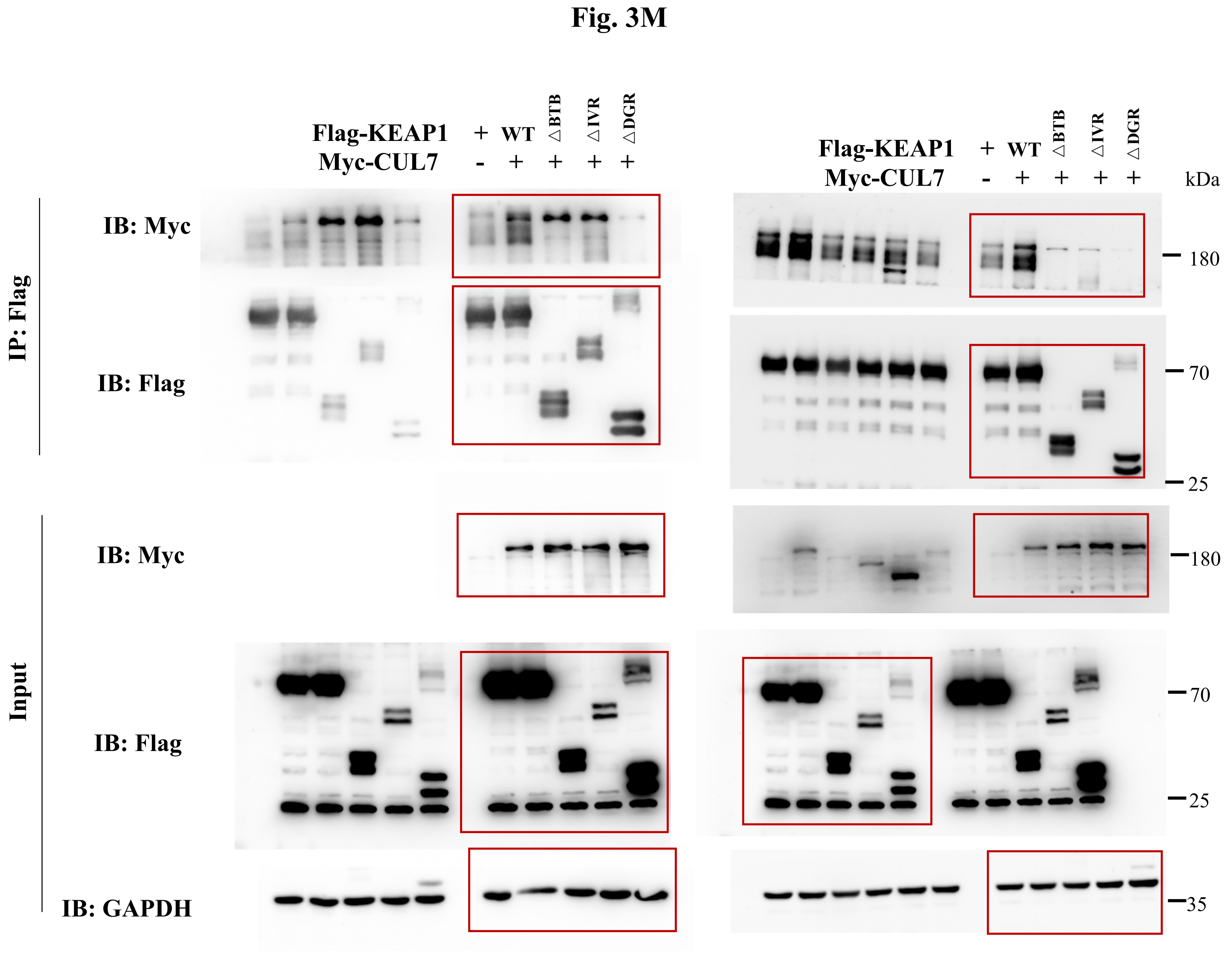

Western blotting images related to Figure 4.

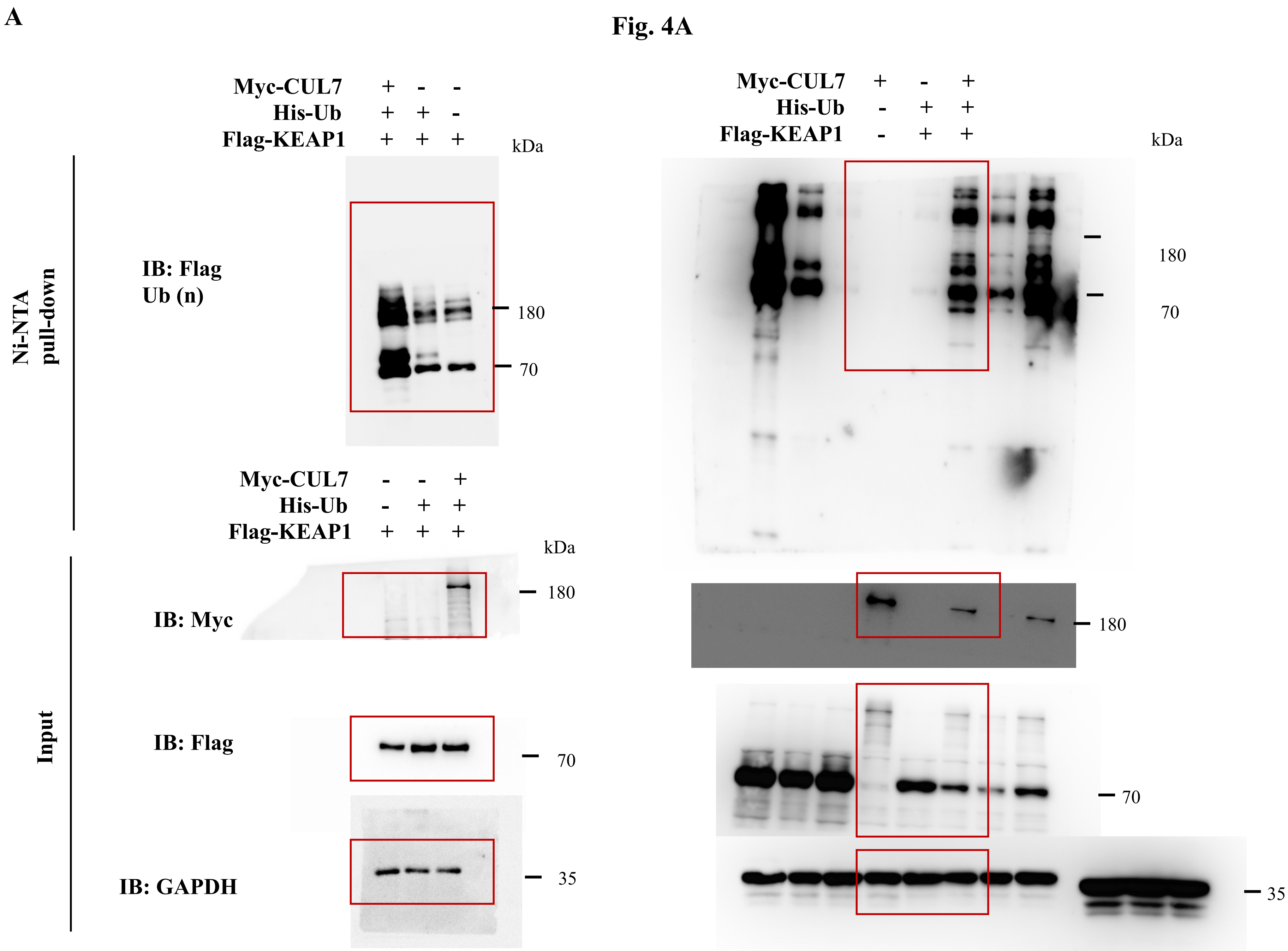

Western blotting images related to Figure 4.

B **Fig. 4B**

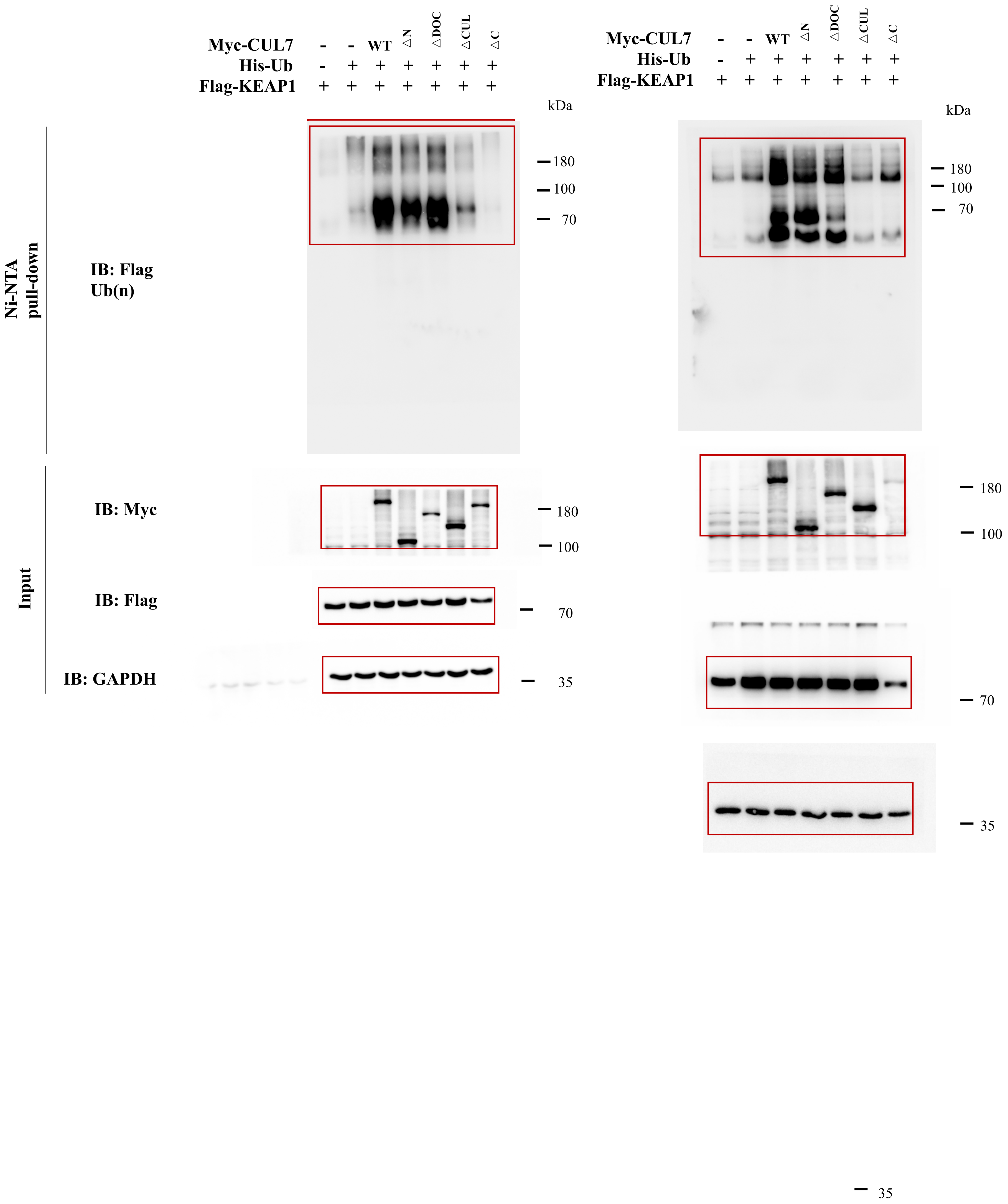

Western blotting images related to Figure 4.

C

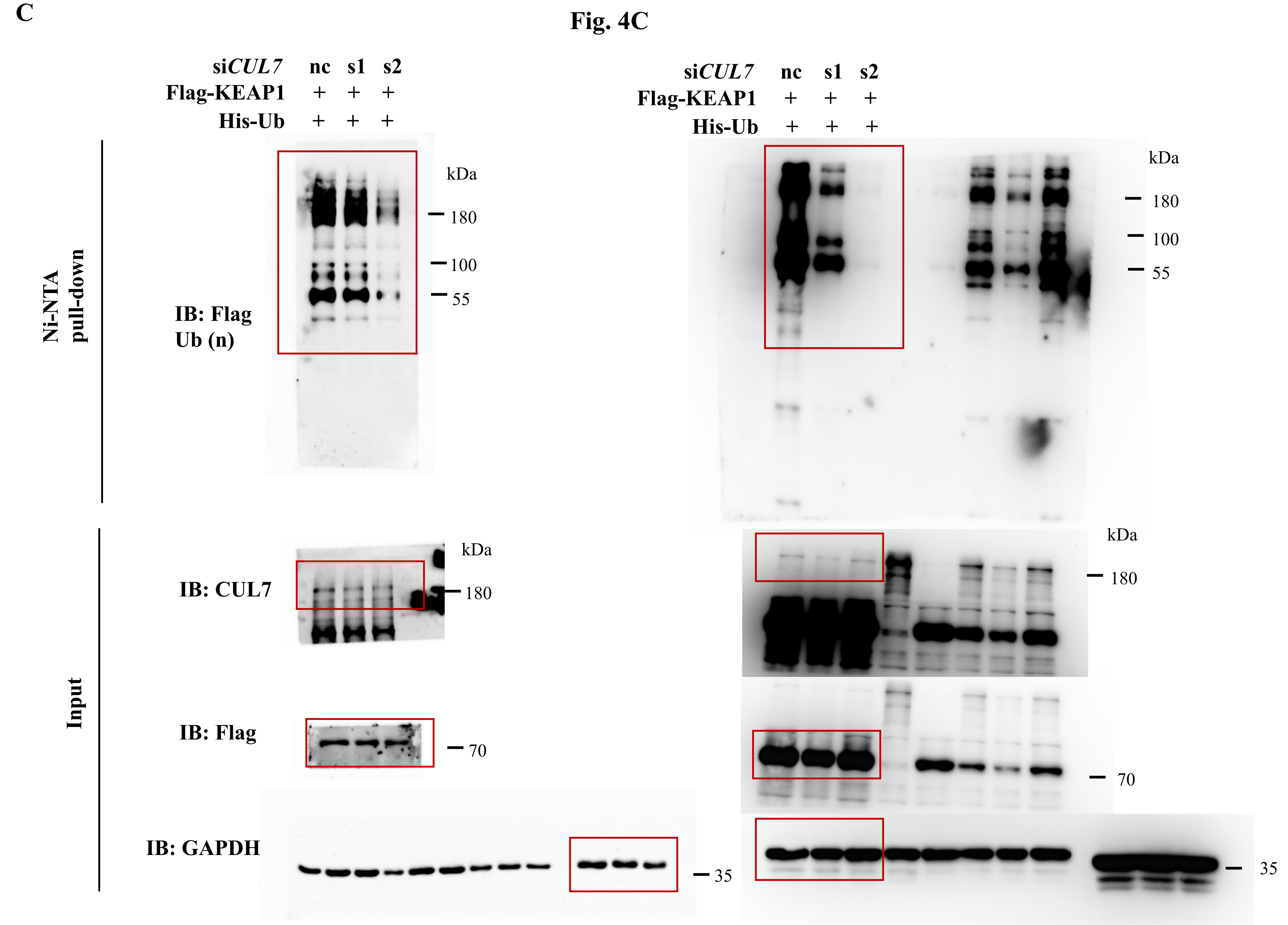

Western blotting images related to Figure 4.

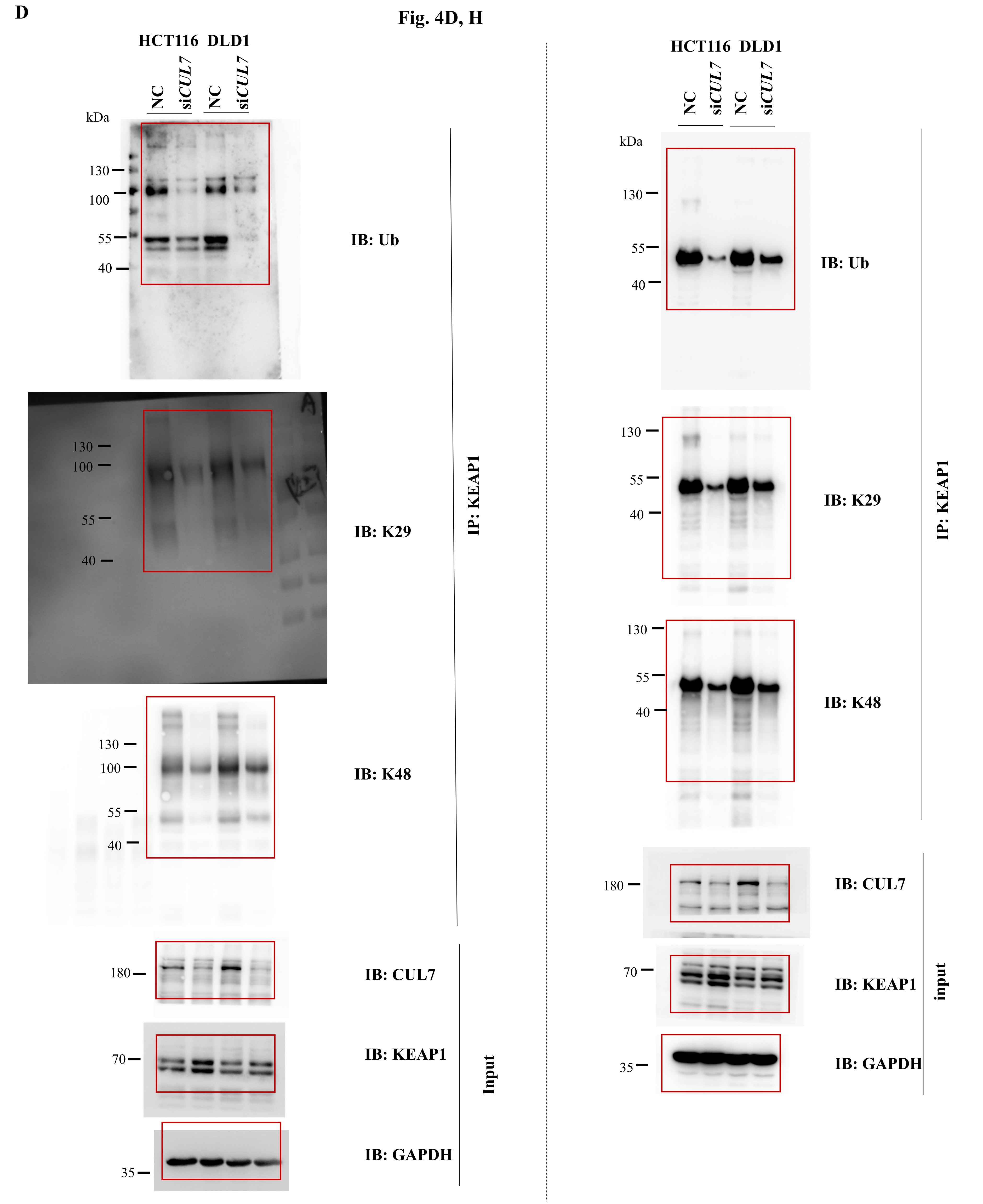

Western blotting images related to Figure 4.

E

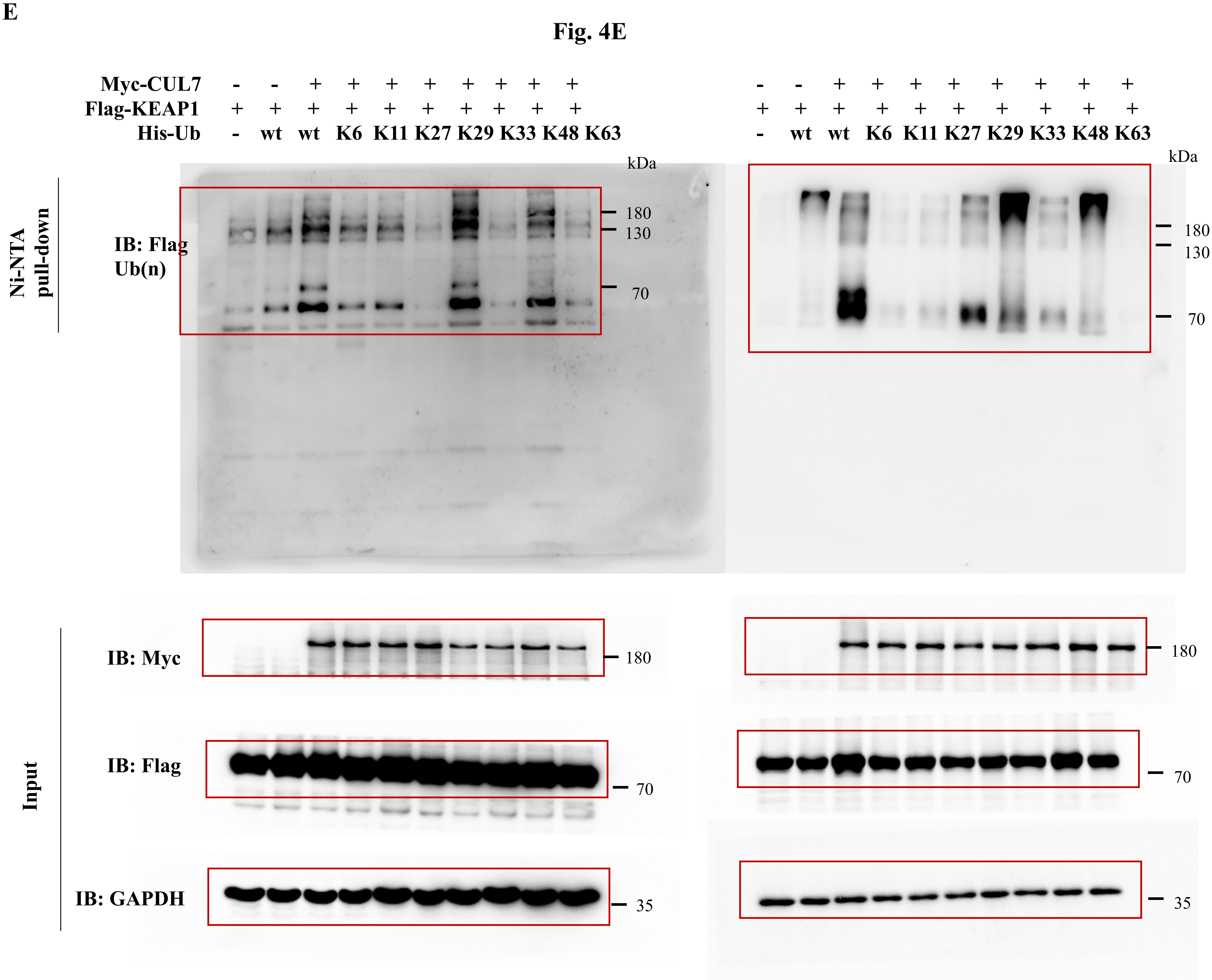

Western blotting images related to Figure 4.

F

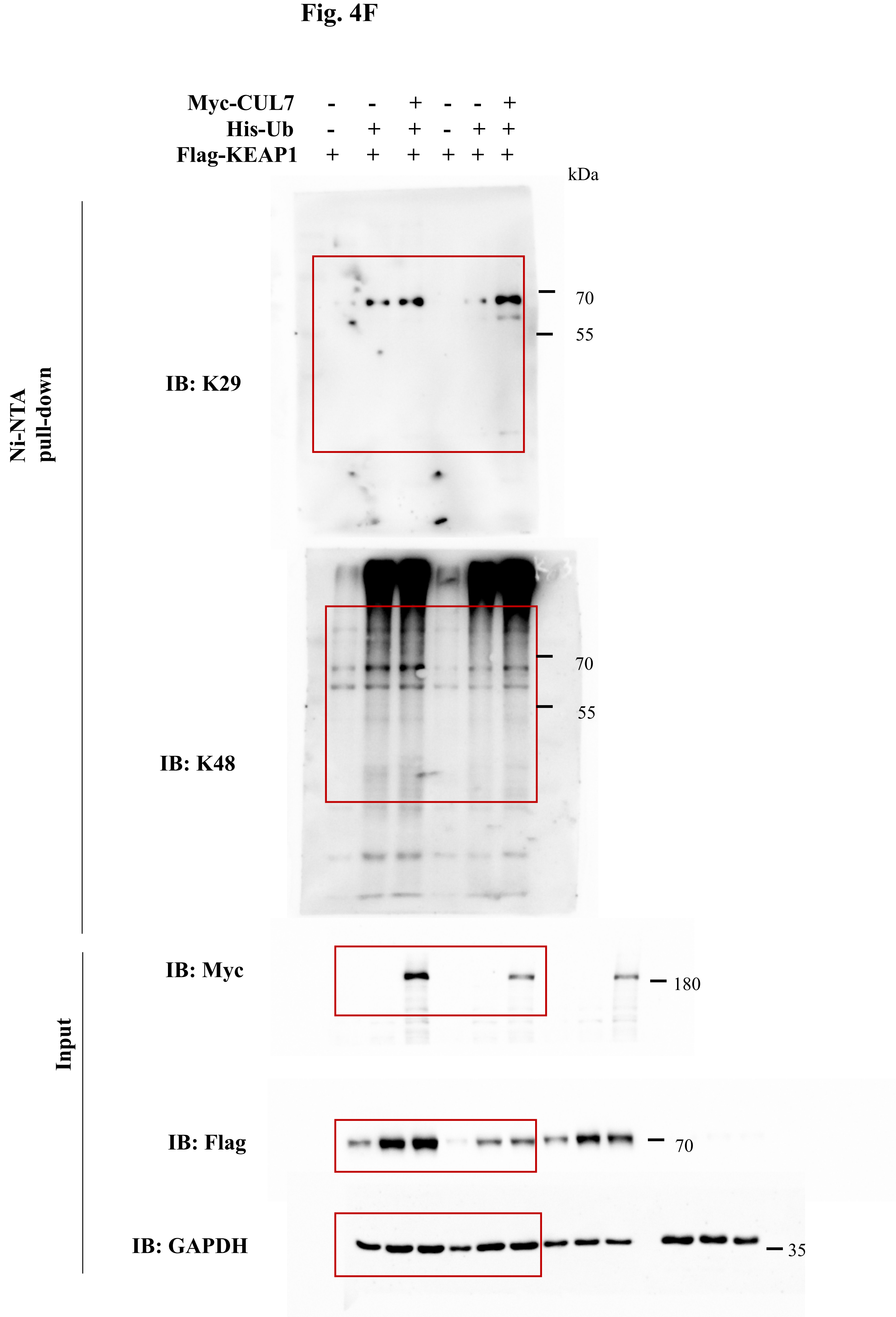

Western blotting images related to Figure 4.

G

Fig. 4G

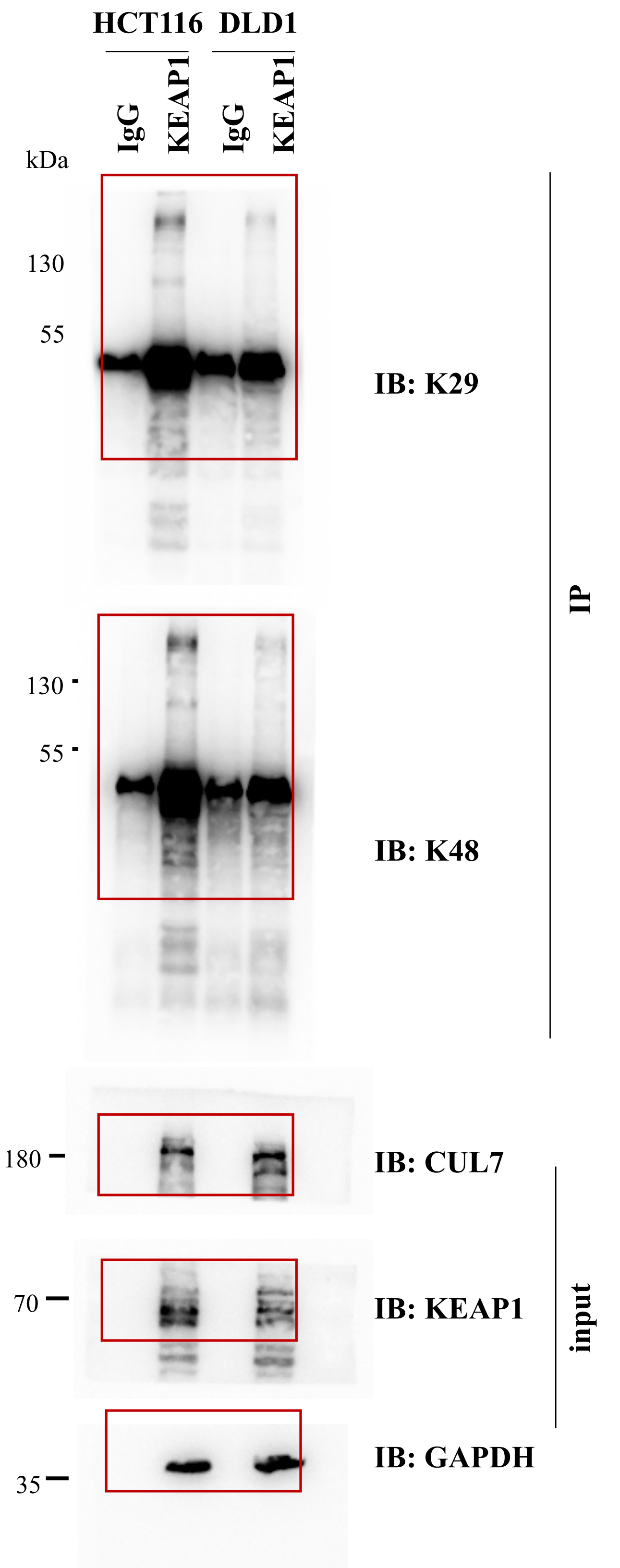

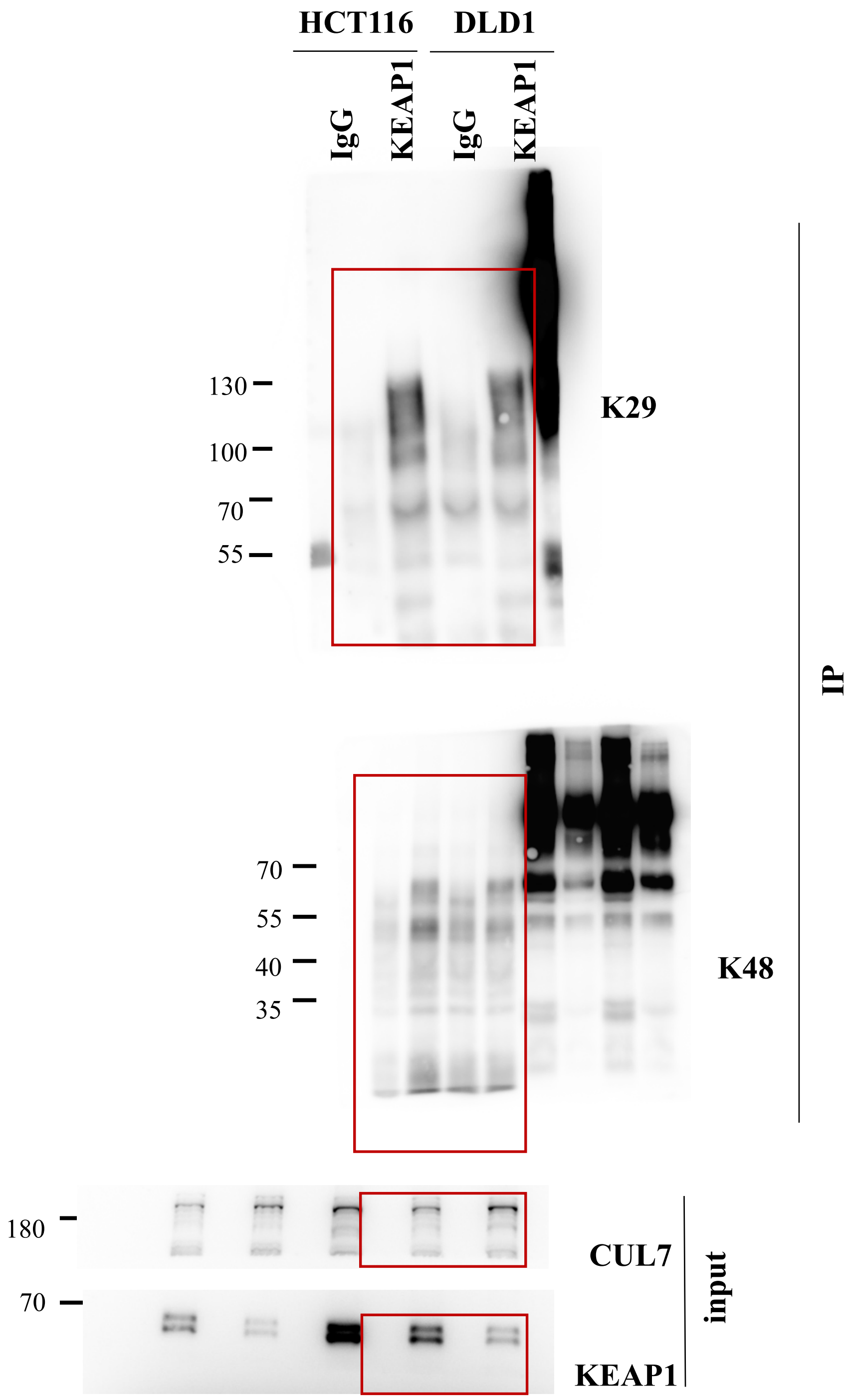

Western blotting images related to Figure 4.

H

Fig. 4I

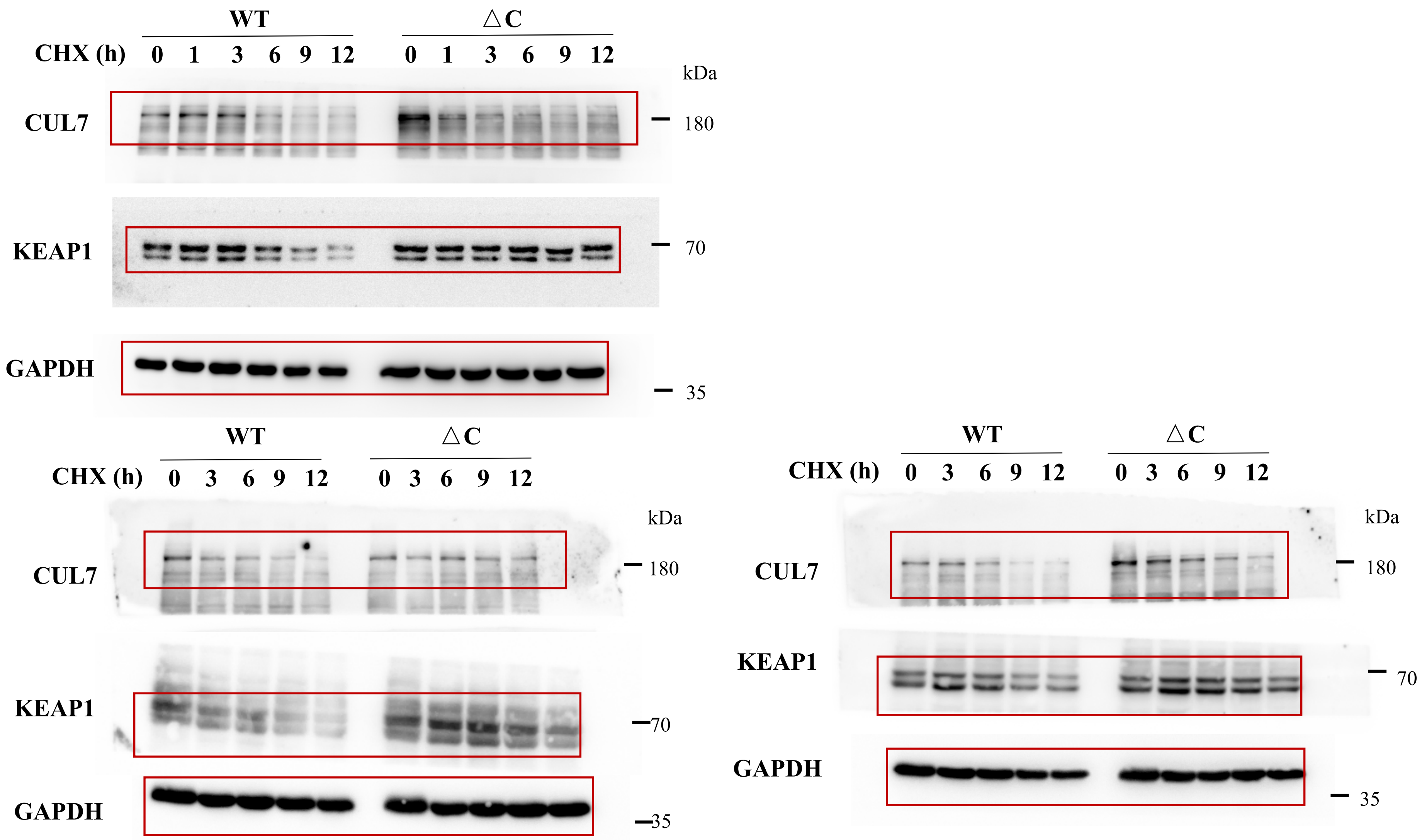

Western blotting images related to Figure 5.

A

Fig. 5A

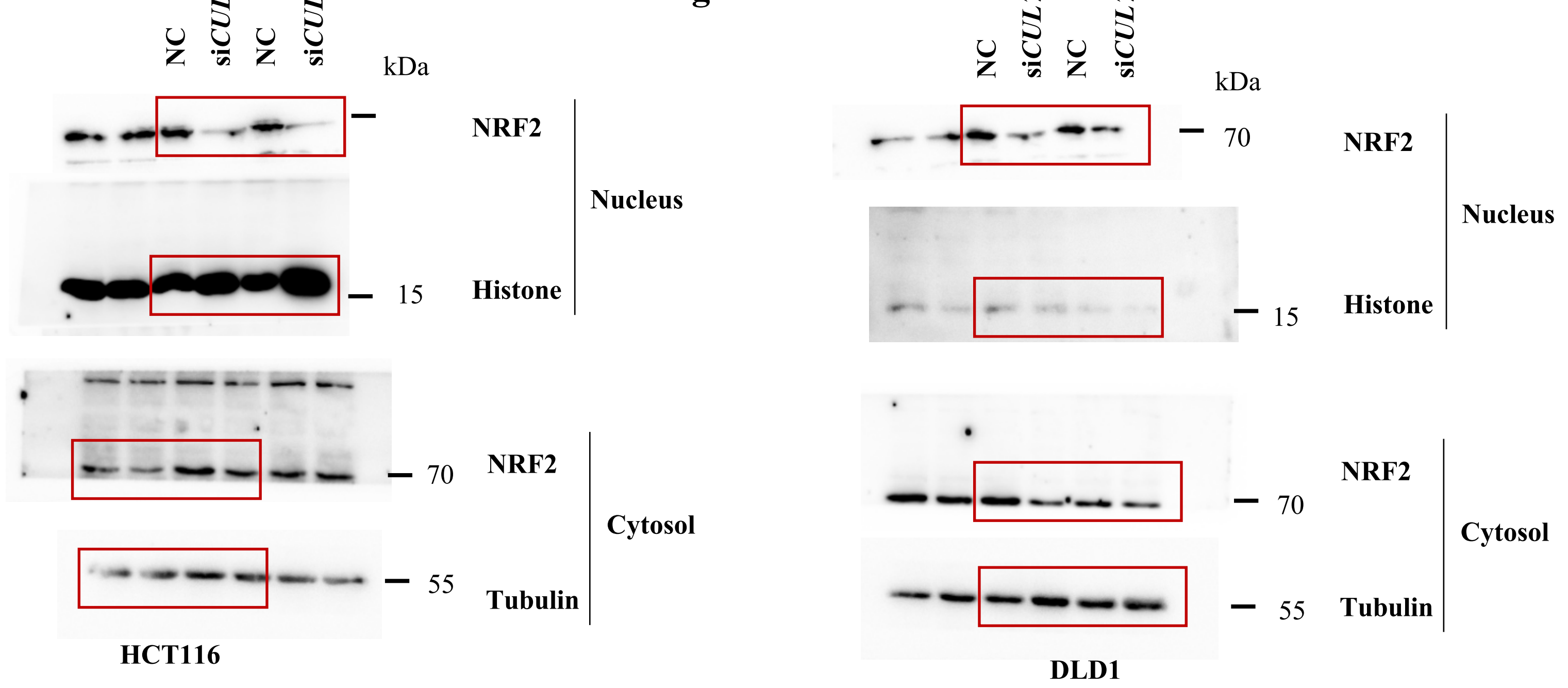

B

Fig. 5B

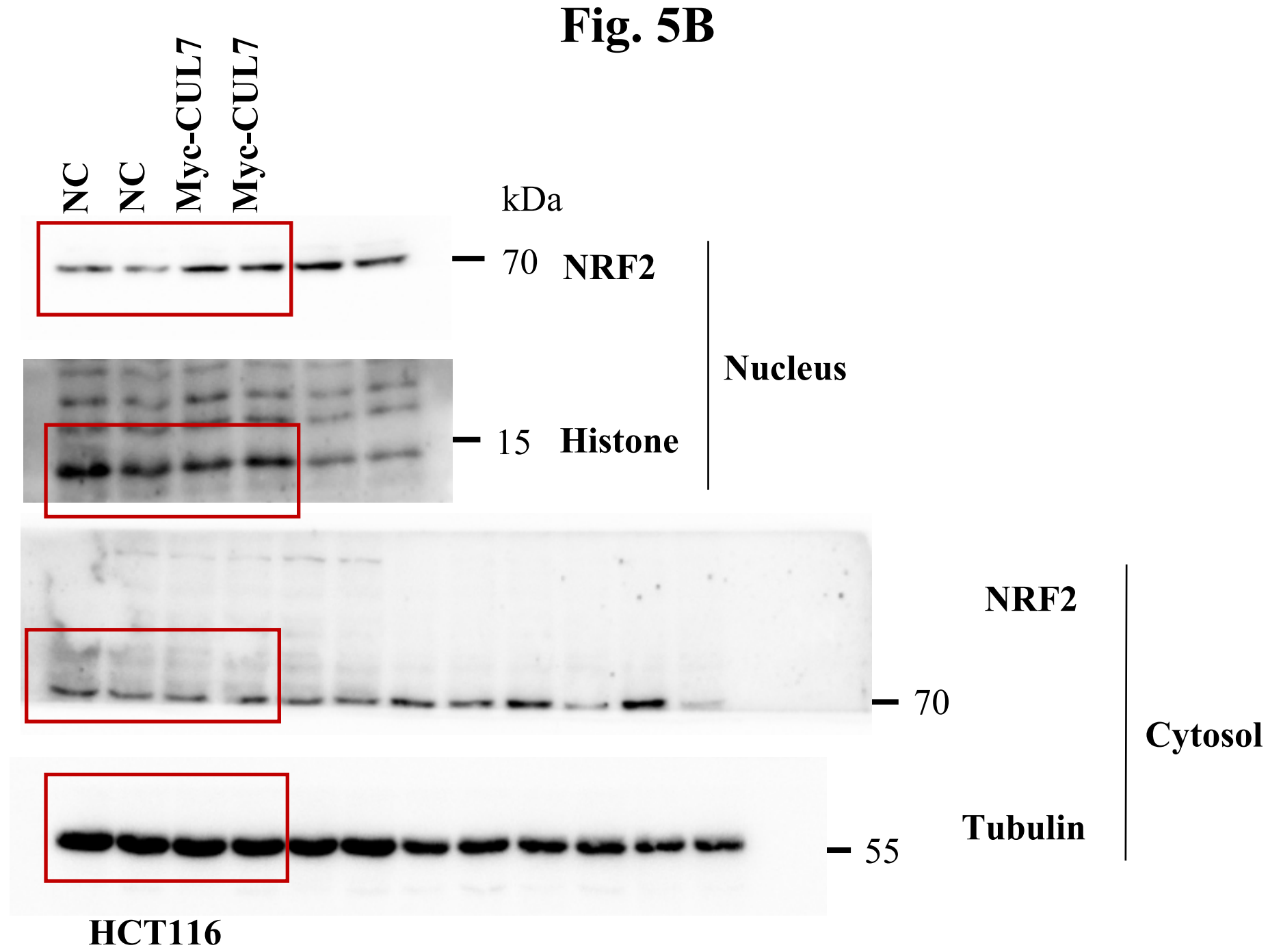

Western blotting images related to Figure 5.

C

Fig. 5H

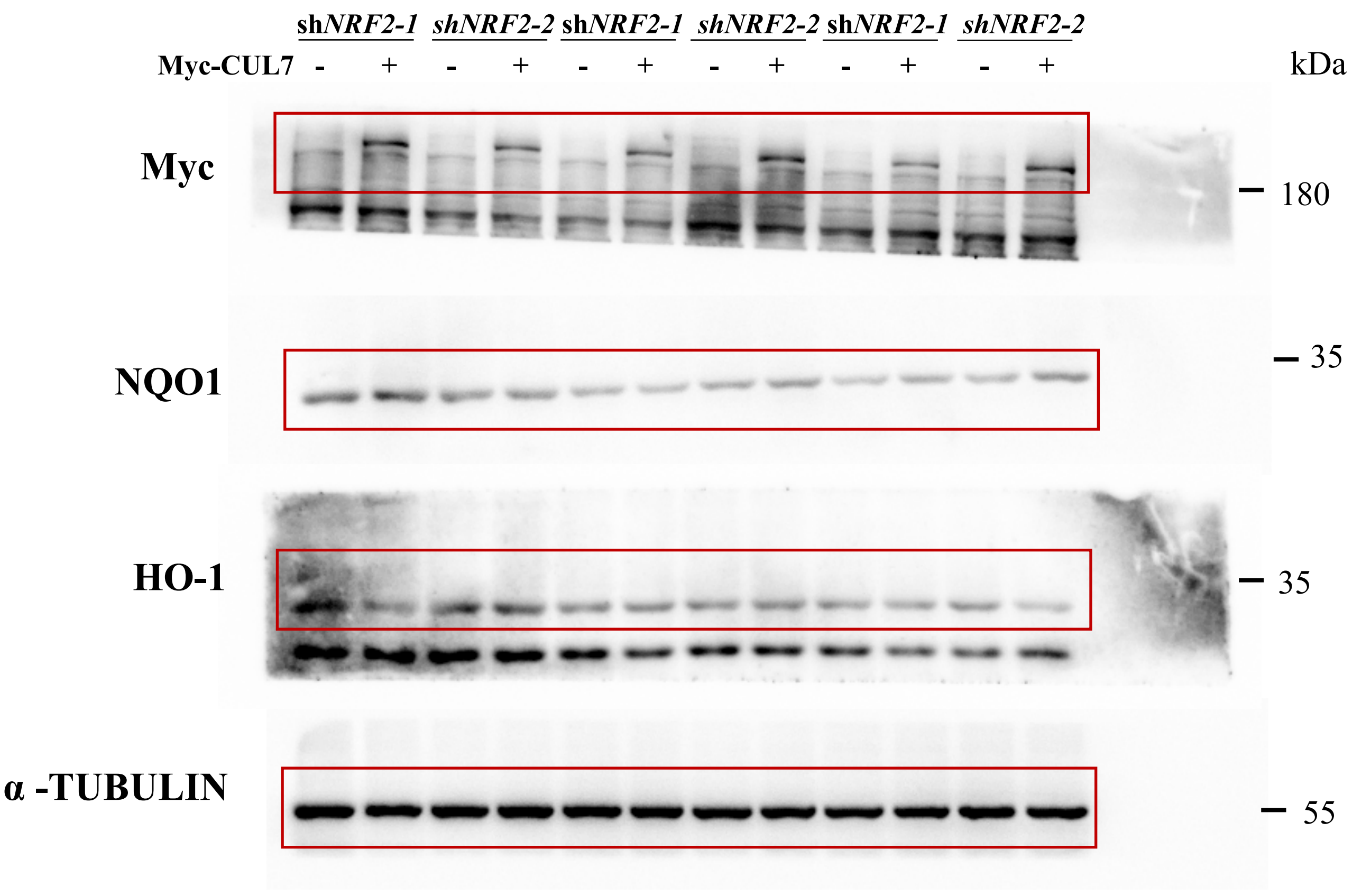

D

Fig. 5M

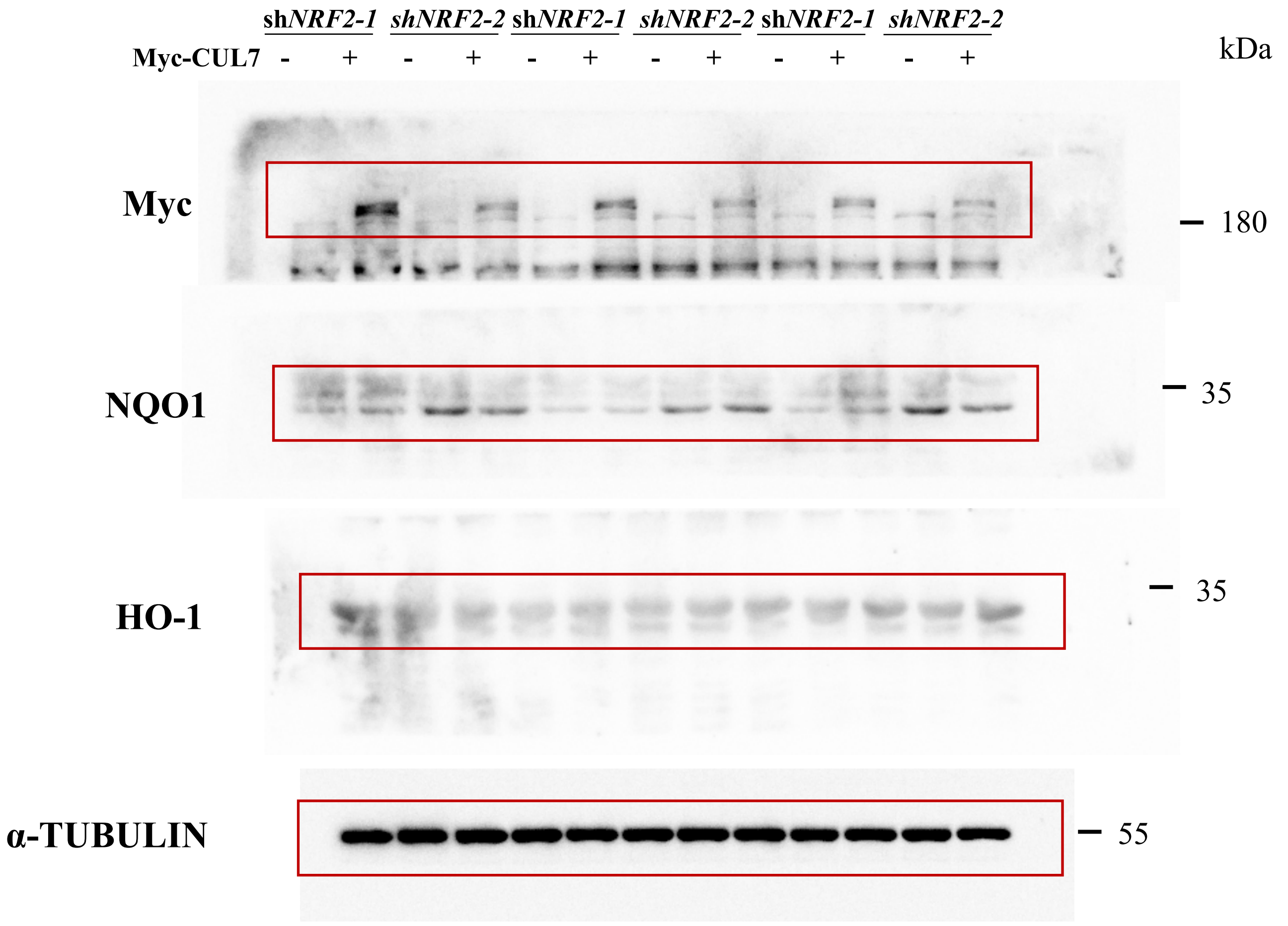

Western blotting images related to Figure 6.

A

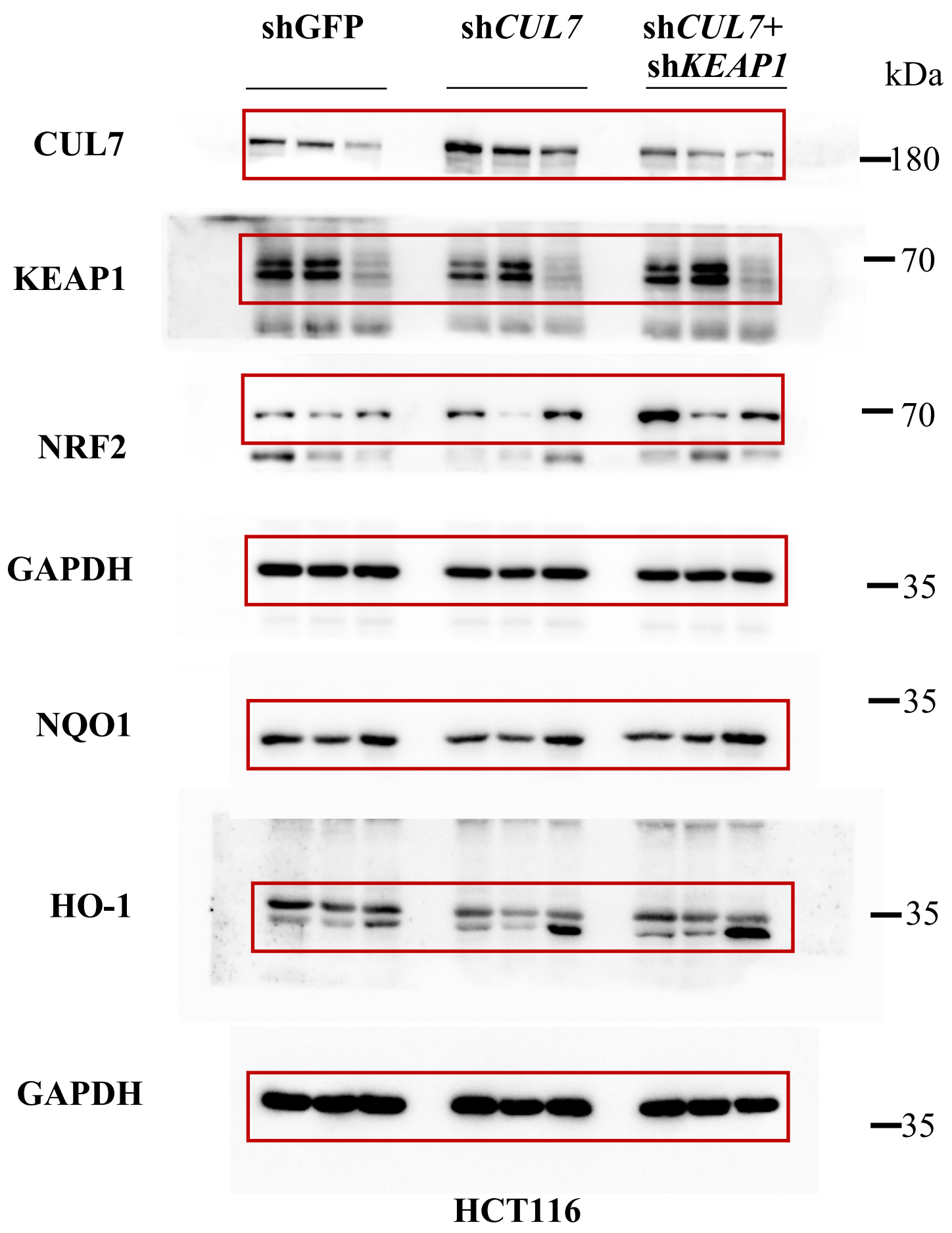

Fig. 6I

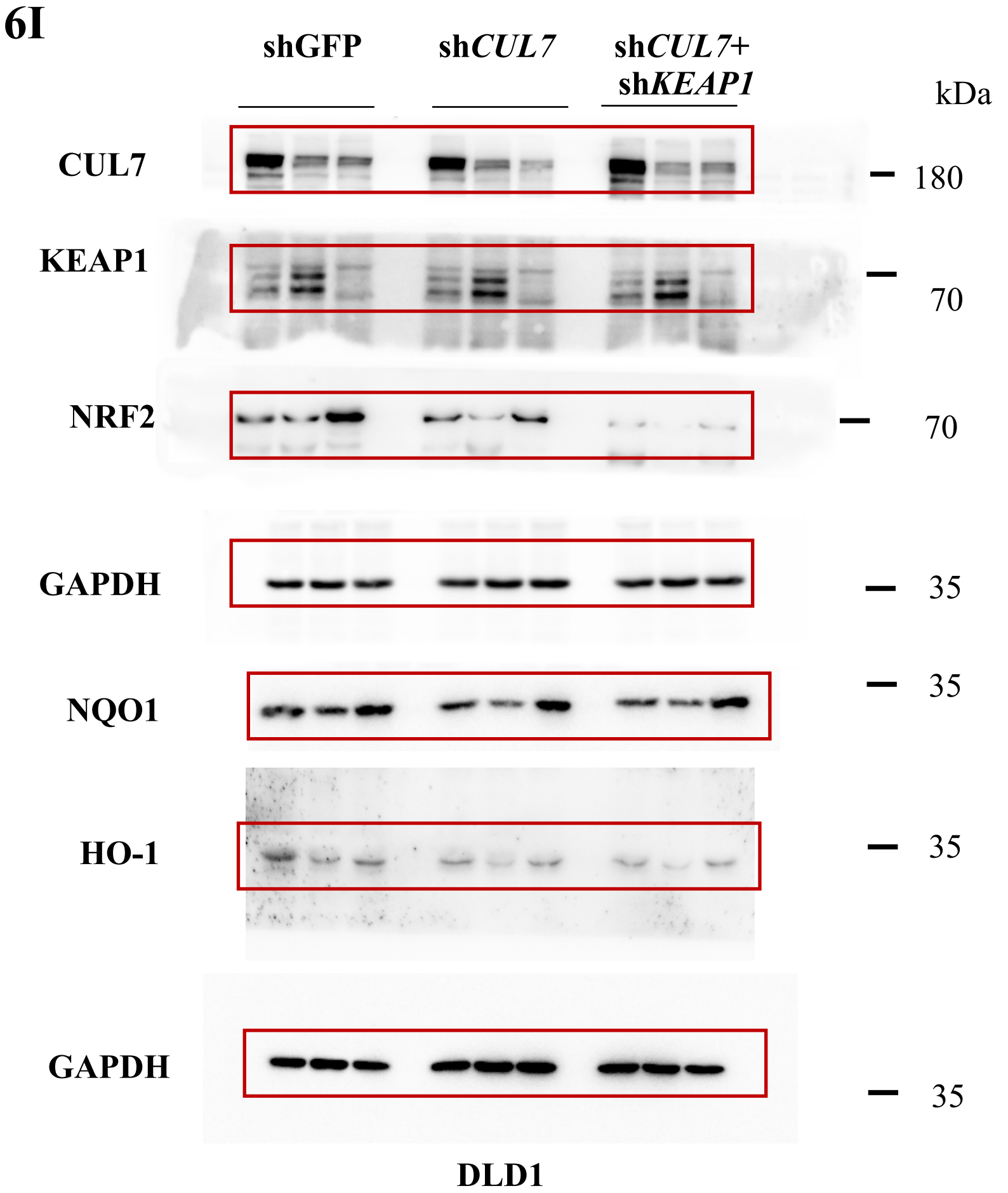

Supplement: Supplementary file 1 [file 41419_2026_8929_MOESM1_ESM.pdf]
