# Supplementary material for: CUL7-mediated KEAP1 ubiquitination promotes the progression of colon cancer via NRF2 signaling
Source: Cell Death Dis. 2026 Jun 8;17(1):712. doi: 10.1038/s41419-026-08929-1 (PMC13462133; doi:10.1038/s41419-026-08929-1)
Supplement: Supplementary file 2 [file 41419_2026_8929_MOESM4_ESM.pdf]

SPRINGER NATURE

**Corresponding Author Name:** Long Jiangang
**Manuscript Number:** CDDIS-25-718

# Reporting Summary

***Springer Nature wishes to improve the reproducibility of the work that we publish. This checklist is used to ensure good reporting standards and to improve the reproducibility. Please respond completely to all questions relevant to your manuscript. For more information, please read the journal's Guide to Authors.***

☑ **Check here to confirm that the following information is available in the Material & Methods section:**

- **The exact sample size (*n*)** for each experimental group/condition, given as a number, not a range
- **A description of the sample collection** allowing the reader to understand whether the samples represent technical or biological replicates (including how many animals, litters, culture, etc.)
- **A statement of how many times the experiment shown was replicated in the laboratory**
- **Definitions of statistical methods and measures**: For small sample sizes (n<5) descriptive statistics are not appropriate, instead plot individual data points
  - Very common tests, such as *t*-test, simple $\chi^2$ tests, Wilcoxon and Mann-Whitney tests, can be unambiguously identified by name only, but more complex techniques should be described in the methods section
  - Are tests one-sided or two-sided?
  - Are there adjustments for multiple comparisons?
  - **Statistical test results**, e.g., ***P* values**
  - Definition of **'center values'** as **median or mean**;
  - Definition of **error bars as s.d. or s.e.m. or c.i.**

***Please ensure that the answers to the following questions are reported in the manuscript itself. We encourage you to include a specific subsection in the methods section for statistics, reagents and animal models. Below, provide the page number or section and paragraph number.***

## Statistics and general methods

| | **Reported in section/paragraph or page #** |
|---|---|
| 1. How was the sample size chosen to ensure adequate power to detect a pre-specified effect size? (Give section/paragraph or page #) | No formal sample size calculation or power analysis was performed to detect a pre-specified effect size. Clinical specimen sample size (n=12) was based on sample |
| For animal studies, include a statement about sample size estimate even if no statistical methods were used. | For animal studies, sample sizes (n=5–8 per group in the AOM/DSS model; n=5–7 per group in xenograft experiments) were based on pilot experiments and |
| 2. Describe inclusion/exclusion criteria if samples or animals were excluded from the analysis. Were the criteria pre-established? (Give section/paragraph or page #) | No samples or animals were excluded from the analysis. Therefore, inclusion/exclusion criteria were not applied. Sample sizes for each experiment are reported in the Figure legends. |
| 3. If a method of randomization was used to determine how samples/animals were allocated to experimental groups and processed, describe it. (Give section/paragraph or page #) | Randomization was used for the AOM/DSS mouse model and xenograft experiments as described in the 'Animals' section. |
| For animal studies, include a statement about randomization even if no randomization was used. | Randomization was used for the AOM/DSS mouse model and xenograft experiments as described in the 'Animals' |

| | |
|---|---|
| 4. If the investigator was blinded to the group allocation during the experiment and/or when assessing the outcome, state the extent of blinding. (Give section/paragraph or page #) | Blinding was used only for outcome assessment of immunohistochemistry. As stated in the 'Tissue microarray and Immunohistochemistry' section, immunostaining was scored by pathologists in a blinded manner. For all other |
| For animal studies, include a statement about blinding even if no blinding was done. | No blinding was performed for animal studies (Animals section). Blinding was used only for IHC outcome |
| 5. For every figure, are statistical tests justified as appropriate? | Statistical analysis (page 15) and figure legends (page 36-39) |
| Do the data meet the assumptions of the tests (e.g., normal distribution)? | The assumptions of the statistical tests (e.g., normal distribution, homogeneity of variances) were not explicitly |
| Is there an estimate of variation within each group of data? | Yes, variation within each group is estimated by standard deviation (SD) as stated in the 'Statistical analysis' section: |
| Is the variance similar between the groups that are being statistically compared? (Give section/paragraph or page #) | The similarity of variance between groups was not formally tested. Parametric tests (t-test and ANOVA) were applied as described in the 'Statistical analysis' section, without |

## Reagents

| | **Reported in section/paragraph or page #** |
|---|---|
| 6. Report the source of antibodies (vendor and catalog number) | Antibodies and reagents section (page 6 and 7) |
| 7. Identify the source of cell lines and report if they were recently authenticated (e.g., by STR profiling) and tested for mycoplasma contamination | Cell culture, cell transfection and viral infection section (paragraph 1, page 8) |

## Animal Models

| | **Reported in section/paragraph or page #** |
|---|---|
| 8. Report species, strain, sex and age of animals | Animals section (page 5 and 6) |
| 9. For experiments involving live vertebrates, include a statement of compliance with ethical regulations and identify the committee(s) approving the experiments. | Ethical approval (page 31) |

10. We recommend consulting the ARRIVE guidelines (*PLoS Biol.* **8**(6), e1000412,2010) to ensure that other relevant aspects of animal studies are adequately reported.

## Human subjects

| | Reported in section/paragraph or page # |
|---|---|
| 11. Identify the committee(s) approving the study protocol. | Ethical approval was obtained from the Ethics Committee of The First Affiliated Hospital of Xi'an Jiaotong University for |
| 12. Include a statement confirming that informed consent was obtained from all subjects. | Informed consent was obtained from all patients whose tissues were used in this study (Clinical specimens section). |
| 13. For publication of patient photos, include a statement confirming that consent to publish was obtained. | Not applicable. No patient photos are included in this study. |
| 14. Report the clinical trial registration number (at ClinicalTrials.gov or equivalent). | Not applicable. This study is not a clinical trial. |

15. For phase II and III randomized controlled trials, please refer to the CONSORT statement and submit the CONSORT checklist with your submission.

16. For tumor marker prognostic studies, we recommend that you follow the REMARK reporting guidelines.

## Data deposition

| | Reported in section/paragraph or page # |
|---|---|
| 17. Provide accession codes for deposited data. Data deposition in a public repository is mandatory for:<br>a. Protein, DNA and RNA sequences<br>b. Macromolecular structures<br>c. Crystallographic data for small molecules<br>d. Microarray data | No data have been deposited in a public repository. The siRNA and shRNA sequences are provided in Table S1. Proteomics and mass spectrometry data are not publicly deposited; raw data are available from the corresponding author upon reasonable request. |

Deposition is strongly recommended for many other datasets for which structured public repositories exist; more details on our data policy are available in the Guide to Authors. We encourage the provision of other source data in supplementary information or in unstructured repositories such as Figshare and Dryad. We encourage publication of Data Descriptors (see Scientific Data) to maximize data reuse.

| | |
|---|---|
| 18. If computer code was used to generate results that are central to the paper's conclusions, include a statement in the Methods section under "**Code availability**" to indicate whether and how the code can be accessed. Include version information as necessary and any restrictions on availability. | Not applicable. No custom computer code was generated or used for the results central to the conclusions. All statistical analyses were performed using GraphPad Prism 8 (as stated in the 'Statistical analysis' section), and no additional code availability statement is required. |
