# Supplementary material for: CUL7-mediated KEAP1 ubiquitination promotes the progression of colon cancer via NRF2 signaling
Source: Cell Death Dis. 2026 Jun 8;17(1):712. doi: 10.1038/s41419-026-08929-1 (PMC13462133; doi:10.1038/s41419-026-08929-1)
Supplement: Supplementary file 3 [file 41419_2026_8929_MOESM2_ESM.docx]

**Supplementary figures**

**Fig. S1 The expression and clinicopathological characteristics of CUL7 in the TCGA dataset of colon cancer. A** Comparison of CUL7 mRNA level between colon cancer and normal tissues in TCGA database using UALCAN. **B**-**E** CUL7 mRNA level in the TCGA based on individual cancer stages, patient’s age, patient’s gender and nodal metastasis status. **p*＜0.05, ***p*≤0.01. Data were analyzed using two-tailed Student’s *t*-test (**A**) and one-way ANOVA (**B**-**E**). Data were represented as mean ± SD. For **A**-**E**, normal tissue/tumor tissue: n=41/286.

**Fig. S2 CUL7 promotes colon cancer progression in colon cancer cells. A** The expression of CUL7 in NCM460 and different colon cancer cell lines. **B** Western blotting analysis in HCT116 and DLD1 cells with overexpression of CUL7 and quantification of protein levels. **C** MTT assay showed the proliferative capabilities of CUL7 in HCT116 and DLD1 cells. **D** Representative images of colony formation in HCT116 and DLD1 cells and quantification of colony number. **E** Representative images of migrated HCT116 and DLD1 cells and quantification of migrated cells. Scale bars represent 200 μm. **F** Western blotting analysis in HCT116 and DLD1 cells stably expressing the sh*CUL7*. **p*＜0.05, ****p*≤0.001, *****p*≤0.0001. Data were analyzed using two-tailed Student’s *t*-test (**B**, **D**, **E**) or two-way ANOVA (**C**). Data were represented as mean ± SD. n=3 biologically independent samples.

**Fig. S3 Identification of intestine-specific *Cul7* knockout mice and their clinicopathology.** **A**, **B** Transgenic mice were identified by PCR of genomic DNA obtained from tail-tip and colon samples. **C** validation of *Cul7* knockout efficiency in intestinal-specific *Cul7*-deficient mice by using western blotting and quantification of protein levels. **D** Body weight change curves of *Cul7^fl/fl^* and *Cul7^fl/fl, vil-Cre^* mice during AD treatment*.* **E** Clinical scores including stool consistency and bleeding of *Cul7^fl/fl^* and *Cul7^fl/fl, vil-Cre^* mice. Data were analyzed using one-way ANOVA (**C**) or two-way ANOVA (**D**, **E**). Data are represented as mean ± SD. n=5 (*Cul7^fl/fl^*+Water), n=6 (*Cul7^fl/fl^*+AD), n=5 (*Cul7^fl/fl, Vil-cre^*+Water) or n=8 (*Cul7^fl/fl, Vil-cre^*+AD). For A, *Cul7^fl/fl^*, red number; *Cul7^fl/fl, vil-cre^*, red number and yellow background. No. 36 *Cul^7fl/fl, vil-cre^* mouse died on day 57.

**Fig. S4 CUL7 regulates KEAP1 stability. A** Label-free quantitative proteomics analysis with the volcano plots. Black represents non-significant proteins. Green represents down-regulated proteins. Red represents up-regulated proteins. **B** Genomic alterations of KEAP1 in colon cancer by using cBioPortal. **C**, **D** Relative mRNA fold change of *CUL7* and *KEAP1* in HCT116 and DLD1 cells transfected with NC or si*CUL7*. **E** Half-life of Keap1 in DLD1 cells transfected with NC or si*CUL7* and KEAP1 protein abundance was quantified by Quantity One, normalized to GAPDH, and then to the t=0 time point. Cells were treated with 200 μg/mL CHX for the indicated time period prior to harvesting. **p*＜0.05, ***p*≤0.01. Data were analyzed using one-way ANOVA (**C**, **D**) or two-way ANOVA (**E**). Data were represented as mean ± SD. n=3 (**A**, **C**-**E**) biologically independent samples.

**Fig. S5 CUL7 interacts with KEAP1.** **A** CoIP assay analyzes the interaction of endogenous CUL7 and KEAP1 in DLD1 cells. **B** HCT116 cells were fixed and immunostained with anti-CUL7 and anti-KEAP1 antibodies prior to confocal microscopy and quantification of the co-localization between CUL7 and KEAP1. Scale bars represent 20 μm. Manders’ Colocalization Coefficients were chosen for the Co-localization Ratio. Manders’ M1 (M2) represents the proportion of red (green) fluorescence overlapped with green (red) fluorescence in total red (green) fluorescence. n=2 (**A**) or n=4 (**B**) biologically independent samples.

**Fig. S6 CUL7 interacts and ubiquitinates KEAP1 is independent with FBXW8.** **A** CoIP assay analyzes the interaction of endogenous FBXW8 and KEAP1 in HCT116 and DLD1 cells. **B** IB analysis of input and IP derived from 293T cells transfected with indicated constructs. Cells were treated with 10 μM MG132 for 12 h prior to harvesting. **C** IB analysis of input and Ni-NTA pull-down products derived from lysates of 293T cells transfected with the indicated construct. Cells were treated with 10 μM MG132 for 12 h prior to harvesting. n=2 biologically independent samples.

**Fig. S7 CUL3 interacts with, but does not degrade KEAP1 in colon cancer cells. A** IB analysis of input and immunoprecipitates derived from 293T cells transfected with KEAP1 and CUL family members, including CUL1, CUL2, CUL3, CUL4a, CUL4b, and CUL5. The cells were treated with 10 μM MG132 for 12 h prior to harvesting. **B** Western blotting analysis of CUL3 and KEAP1 in HCT116 and DLD1 cells after knockdown of *CUL3* and quantification of protein levels. **p*＜0.05, ***p*≤0.01, ****p*≤0.001. Data were analyzed using one-way ANOVA (**B**). Data were represented as mean ± SD. n=2 (**A**) or n=3 (**B**) biologically independent samples.

**Fig. S8 NRF2 mediates the effect of CUL7 on cell growth of colon cancer.** **A** Western blotting analysis of NRF2 in HCT116 cells depleted of *NRF2* by using si*NRF2* and quantification of protein levels. **B**, **C** Western blotting analysis of NRF2 in HCT116 and DLD1 cells stably depleted of *NRF2* by using sh*NRF2*. **D** Relative mRNA fold change of antioxidant genes, including *NQO1* and *HO-1* in *NRF2*-depleted DLD1 cells expressing EV or Myc-CUL7. **E** Western blotting analysis of NQO1, HO-1 and Myc-tag in *NRF2*-depleted DLD1 cells expressing EV or Myc-CUL7 and quantification of protein levels. **F** Relative ROS levels in *NRF2*-depleted DLD1 cells expressing EV or Myc-CUL7. **G** Representative MTT analysis of cell growth in *NRF2*-depleted DLD1 cells expressing EV or Myc-CUL7. **H** Representative images of colony formation and quantification of colony number in *NRF2*-depleted DLD1 cells expressing EV or Myc-CUL7. Data were analyzed using two-tailed Student’s t-test (**A**, **D-F**, **H**) and two-way ANOVA (**G**). Data were represented as mean ± SD. n=3 biologically independent samples.

**Fig. S9 Validation of *CUL7* and *KEAP1* knockdown efficiency and tumor histology. A** Western blotting analysis of CUL7 and KEAP1 in HCT116 and DLD1 cells with simultaneously knockdown of *CUL7* and *KEAP1* and quantification of protein levels. **B** Western blotting analysis of CUL7 and KEAP1 in HCT116 and DLD1 cells stably expressing sh*CUL7* and sh*KEAP1*. **C** Representative images of HE staining in indicated HCT116 and DLD1 tumors of nude mice. Scale bars represent 100 μm. **p*＜0.05, ***p*≤0.01, ****p*≤0.001. Data were analyzed using one-way ANOVA (**A**). Data were represented as mean ± SD. n=3 biologically independent samples.

**Fig. S10 The expression and overall survival of NQO1 in colon cancer and the correlation between CUL7 and NQO1.** **A** Comparison of NQO1 mRNA level between colon cancer and normal tissues in TCGA database using GEPIA2 (normal tissue/tumor tissue: n=349/275). **B** The correlation between CUL7 and NQO1 in colon cancer (n=275). **C** Kaplan-Meier analysis of percent survival of NQO1 in colon cancer patients (colon cancer patients with low or high expression of NQO1: n=544/517). Data were analyzed using two-tailed Student’s *t*-test (**A**), Spearman correlation analysis (**B**) and two-side log-rank test (**C**). Data were represented as mean ± SD.
