# Supplementary material for: CUL7-mediated KEAP1 ubiquitination promotes the progression of colon cancer via NRF2 signaling
Source: Cell Death Dis. 2026 Jun 8;17(1):712. doi: 10.1038/s41419-026-08929-1 (PMC13462133; doi:10.1038/s41419-026-08929-1)
Supplement: Supplementary file 4 [file 41419_2026_8929_MOESM3_ESM.docx]

**Supplementary tables**

**Table S1.** Sequence of siRNA and shRNA.

| Genes | Sequence of shRNA |
| --- | --- |
| Negative Control | TTCTCCGAACGTGTCACGT |
| si*CUL7* (s1) | GCACAAGAGCGAGAAGGAA |
| si*CUL7* (s2) | TGAGATCCTAGCTGAACTG |
| si*KEAP1* | GTCCTGCACAACTGUATCT |
| si*NRF2* (s1) | CGCTCAGTTACAACTAGAT |
| si*NRF2* (s2) | GAGACTACCATGGTTCCAA |
| si*CUL3* (s1) | GCACATGAAGACTATAGTA |
| si*CUL3* (s2) | CGGCAAACTCTATTGGATA |
| shGFP | GCAAGCTGACCCTGAAGTTCAT |
| sh*CUL7* | TGAGATCCTAGCTGAACTG |
| sh*KEAP1* | GTCCTGCACAACTGUATCT |
| sh*NRF2* (sh1) | AGTTTGGGAGGAGCTATTATC |
| sh*NRF2* (sh2) | CCGGCATTTCACTAAACACAA |

**Table S2.** The primers used in the study.

| Gene Name | Primers sequences (5’- 3’) | Primers length (bp) |
| --- | --- | --- |
| Human-*CUL7* | Forward: CCATCTCAGAGTCCCAACAC  Reverse: TTCAGCACCACGGCATAG | 20  18 |
| Human-*KEAP1* | Forward: TCCCCTACAGCCAAGGTCC  Reverse: TCAGTGGAGGCGTACATCAC | 19  20 |
| Human-*NQO1* | Forward: CCTTCCGGAGTAAGAAGGCAG  Reverse: TCCAGGCGTTTCTTCCATCC | 21  20 |
| Human-*HO-1* | Forward: ATGACACCAAGGACCAGAGC  Reverse: GCATAAAGCCCTACAGCAACT | 20  21 |
| Human-*GAPDH* | Forward: CAGGAGGCATTGCTGATGAT  Reverse: GAAGGCTGGGGCTCATTT | 20  18 |
| Mus-*Flox* | Forward: ACACACCCGTTTCCCTAAATTAC  Reverse: GAACATTGCGGAGTTTGTTATTC | 23  23 |
| Mus-*Cre* | Forward: GAACGCACTGATTTCGACCA  Reverse: GCTAACCAGCGTTTTCGTTC | 20  20 |

**Table S3.** Differential expression of CUL7 in colon cancer and adjacent tissues.

|  | n | CUL7 expression | | Chi-square  Value | p value |
| --- | --- | --- | --- | --- | --- |
|  |  | High | Low |  |  |
| Colon cancer | 92 | 34 | 58 | 26.02 | 0.0001*** |
| Adjacent tissues | 75 | 3 | 72 |  |  |

P values were determined by the Chi-square. * * * Statistically significant (*p* ＜ 0.001)

**Table S4.** Correlation between CUL7 expression and clinicopathological characteristics.

|  | variables | CUL7 expression | | total | x^2^ | p value |
| --- | --- | --- | --- | --- | --- | --- |
|  |  | Low | High |  |  |  |
| Age (year) |  |  |  |  | 1.215 | 0.2704 |
|  | ＜60 | 20 | 8 | 28 |  |  |
|  | ≥60 | 38 | 26 | 64 |  |  |
| Sex |  |  |  |  | 0.7465 | 0.3876 |
|  | Female | 27 | 19 | 46 |  |  |
|  | Male | 31 | 15 | 46 |  |  |
| Grade |  |  |  |  | 8.403 | 0.0037* |
|  | I/II | 53 | 23 | 76 |  |  |
|  | III | 5 | 11 | 16 |  |  |
